# Life-course body size trajectories and the progression of cardio-renal-metabolic multimorbidity: a prospective UK biobank study

**DOI:** 10.1186/s12986-026-01148-7

**Published:** 2026-06-04

**Authors:** Jingjing Lang, Zhongyang Guan, Lili Li, Lu Jiang, Guanghui Mao, Guochong Chen, Mario Siervo, Xiaoyan Shi

**Affiliations:** 1 Children’s Hospital of Soochow University, Suzhou, Jiangsu China; 2 Faculty of Health Sciences, Curtin School of Population Health, Curtin University, Perth, WA Australia; 3 Dementia Centre of Excellence, Faculty of Health Sciences, Curtin enAble Institute, Curtin University, Perth, WA Australia; 4 Department of Nutrition and Food Hygiene, School of Public Health, Suzhou Medical College of Soochow University, Suzhou, Jiangsu China

**Keywords:** Obesity, Body size trajectory, Cardio-renal-metabolic multimorbidity, Cardiovascular disease, Type 2 diabetes, Chronic kidney disease, Multi-state model

## Abstract

**Background:**

The influence of life-course body size on the progression of cardio-renal-metabolic multimorbidity (CRMM) is insufficiently understood.

**Methods:**

We included 398,491 UK Biobank participants. Childhood-to-adulthood body size trajectories were defined using recalled childhood somatotype and adult body mass index (BMI). Multi-state models evaluated transition-specific hazards, state-occupation probabilities, and expected length of stay (ELOS) across body size trajectories. Progression was represented as a comorbidity-count pathway (healthy, first/double/triple CRMM, and death) and a comorbidity-pattern pathway specifying the entry disease and comorbidities among cardiovascular disease (CVD), type 2 diabetes (T2D), and chronic kidney disease (CKD). Subgroup and interaction analyses evaluated joint effects of trajectory and Life’s Essential 4 (LE4). Mediation by the C-reactive protein-triglyceride-glucose index (CTI) was assessed.

**Results:**

Trajectories culminating in obesity were associated with higher hazards along the comorbidity-count pathway and shorter healthy time (ELOS 13.08 vs. 10.59 years for Average-to-normal vs Thinner-to-obesity). Comorbidity-pattern analyses identified T2D-containing comorbidities (T2D-CKD, T2D-CVD) as hubs toward CRMM. Compared with the Average-to-normal group, hazard ratios (HRs) were highest when the destination included T2D: CVD to T2D-CVD, 3.24 (Plumper-to-obesity), and 3.85 (Thinner-to-obesity); CKD to T2D-CKD, 2.66 (Plumper-to-obesity) and 4.03 (Thinner-to-obesity). Moving from LE4-low to LE4-high attenuated the risks of CRMM associated with progressive or persistent obesity trajectories by 26.8-37.8%. CTI mediated approximately 42-54% of the associations between sustained or progressive obesity trajectories and CRMM comorbidity outcomes.

**Conclusions:**

Progressive or persistent obesity trajectories were associated with higher CRMM progression hazards. Early adiposity prevention and sustained lifestyle improvement may slow this progression. Integrating body size trajectory and lifestyle factors with metabolic inflammation indicators may strengthen risk stratification.

**Supplementary Information:**

The online version contains supplementary material available at 10.1186/s12986-026-01148-7.

## Introduction

As global population aging intensifies, approximately one in every three deaths worldwide is attributable to cardiovascular disease (CVD), type 2 diabetes (T2D), or chronic kidney disease (CKD) [1]. These conditions share common pathophysiological mechanisms, including hyperglycemia-driven mitochondrial superoxide overproduction and oxidative stress, activation of the renin-angiotensin-aldosterone system (RAAS), endothelial dysfunction and reduced nitric oxide production, and insulin resistance-related lipid overload and lipotoxicity [2–5]. These mechanisms are closely correlated and over time they may amplify one another, increasing their likelihood of clustering and multimorbidity [2, 6]. Cardio-renal-metabolic multimorbidity (CRMM), defined as the concomitant diagnosis of CVD, T2D, and CKD in the same individual, represents a growing global health burden. Previous studies have suggested that CRMM may synergistically increase mortality risk and reduce life expectancy compared to each condition alone [7–10]. Health-care utilization and costs escalate markedly when any new cardio-renal-metabolic condition is diagnosed [11], which highlight the need to understand the patterns of onset of CRMM and identify modifiable targets for early prevention and effective interventions.

Obesity in both childhood and adulthood is a major risk factor for CRMM [4, 5]. Previous studies have mainly focused on the associations between adult obesity and CRMM or its component diseases, largely relying on cross-sectional or single time-point measures of adiposity [12], thus failing to capture the cumulative exposure or timing of excess weight across the life course. Childhood-to-adulthood body size trajectories capture the timing, direction, and persistence of weight change, thereby approximating the dose and duration of excess adiposity more closely than a single adult body mass index (BMI) measure [13]. Distinguishing stable normal weight, adult-onset obesity, and persistent or progressive obesity could help to identify heterogeneity in CRMM risk and clarifies prevention windows and intervention priorities [13–16].

Meanwhile, most existing studies have focused on static comorbidity states, overlooking the dynamic and sequential nature of multimorbidity progression [16]. In settings where individuals may transition from healthy to a first cardiometabolic disease, then to double and triple CRMM, and ultimately to death, such approaches cannot capture when, how fast, and through which routes progression to each health state may occur. Multi-state models overcome this limitation by modeling transitions between health states, and estimating transition-specific hazards, probabilities, and expected length of stay (ELOS) [17, 18]. The application of these frameworks to childhood-to-adulthood body size trajectories may enable the identification of individuals who are more likely to exit the healthy state earlier, to accumulate additional conditions more rapidly, and to remain longer durations in multimorbid states, thereby highlighting prevention windows and intervention targets along the CRMM pathway.

In addition to body size trajectories alone, modifiable behavioral and clinical factors may further shape the development of CRMM. Healthy lifestyle metrics, such as Life’s Essential 8 (LE8), are widely used to capture such modifiable factors [19]. Building on this framework, cross-classifying body size trajectories with lifestyle metrics may enhance risk stratification and quantify the extent to which adherence to healthy lifestyle behaviors mitigates the risks associated with adverse trajectories [20]. Moreover, biological mechanisms linking excess adiposity to CRMM remain incompletely understood. Chronic low-grade inflammation and insulin resistance are key mediating pathways through which obesity contributes to metabolic, vascular, and renal dysfunction [21, 22]. The C-reactive protein-triglyceride-glucose index (CTI) has recently emerged as a composite marker reflecting both inflammation and metabolic health [23]. However, the extent to which CTI mediates the associations between body size trajectories and CRMM remains unclear. Addressing this knowledge gap may clarify the underlying mechanisms and help prioritize anti-inflammatory and insulin-sensitizing prevention strategies.

**Fig. 1:**
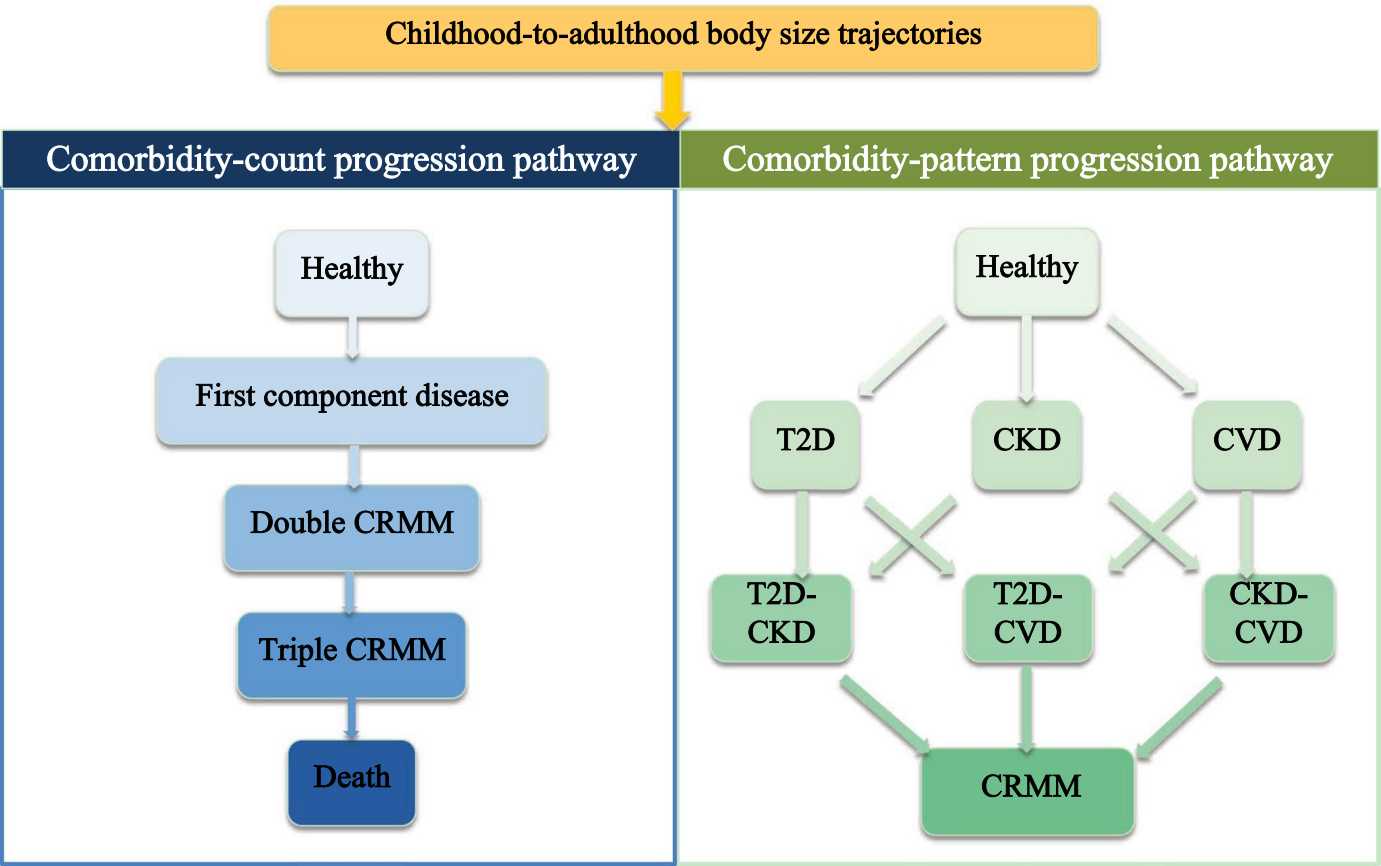
Conceptual framework of CRMM progression pathways. The figure illustrates two complementary pathways of cardiometabolic multimorbidity progression examined in this study: a comorbidity-count progression pathway representing the sequential accumulation of diseases (Healthy → First component disease → Double CRMM → Triple CRMM → Death), and a comorbidity-pattern progression pathway representing specific transition patterns among T2D, CKD, and CVD, including intermediate combinations (T2D-CKD, T2D-CVD, CKD-CVD) leading to CRMM. Childhood-to-adulthood body size trajectories are hypothesized to influence both pathways. Abbreviations: CRMM, cardio-renal-metabolic multimorbidity; T2D, type 2 diabetes; CKD, chronic kidney disease; CVD, cardiovascular disease

We investigated whether childhood-to-adulthood body size trajectories are associated with CRMM incidence and mortality in a large prospective cohort. To illustrate the analytical framework, we used two multi-state approaches, comorbidity-count and comorbidity-pattern, to quantify transition hazards and expected length of stay (ELOS) from the healthy state to CRMM and death across body size trajectories (Fig. 1). We further examined whether lifestyle refines risk stratification within trajectories and whether CTI mediates these associations.

## Methods

### Study design and population

The UK Biobank is an ongoing prospective cohort across the United Kingdom that recruited 502,364 adults aged 40 to 69 years between 2006 and 2010 [24]. Baseline assessments combined touch-screen questionnaires, nurse interviews, physical measurements, and biospecimen assays. Health outcomes are updated through linkage to national electronic health records. The UK Biobank obtained ethics approval, and all participants provided written informed consent. For this study, we limited the cohort to participants free of CVD, T2D, or CKD at baseline. After excluding 87,737 with prevalent disease, 414,627 participants remained (Figure S1). We further excluded participants with missing data on childhood body shape or adult BMI (n=16,092), as well as those missing information on all healthy lifestyle metrics (n=44). The final analytical sample comprised 398,491 participants.

### Definitions of childhood-to-adulthood body size trajectories

Childhood body size was assessed at baseline via a touchscreen item in which participants rated their size at age 10 relative to peers with three options: “thinner,” “about average,” or “plumper.” Responses of “do not know” or “prefer not to answer” were considered as missing and excluded. In adulthood, weight and height were obtained by trained staff following standardized procedures. BMI was calculated as weight (kg) divided by height squared (m^2^) and categorized as normal weight (<25.0 kg/m^2^), overweight (25.0–29.9 kg/m^2^), or obesity (≥30.0 kg/m^2^) [25]. Combining the childhood category with the adult BMI category yielded nine childhood-to-adulthood body size trajectories: Thinner-to-Normal, Thinner-to-Overweight, Thinner-to-Obesity, Average-to-Normal, Average-to-Overweight, Average-to-Obesity, Plumper-to-Normal, Plumper-to-Overweight, and Plumper-to-Obesity.

### Assessment of the outcomes

Health outcomes were ascertained via linkage to hospital inpatient and death registry data. Follow-up time was calculated from baseline to the earliest occurrence of an outcome event, death, loss to follow-up, or the last available health record. Primary outcomes included incident CVD, T2D, CKD, first component disease, double CRMM, the three pairwise comorbidities (T2D-CVD, CVD-CKD, T2D-CKD), triple CRMM, and all-cause mortality. CVD was defined using International Statistical Classification of Diseases and Related Health Problems, 10th edition (ICD-10) diagnostic codes I20-I25 (coronary heart disease) and I60-I64 (stroke). T2D was identified by ICD-10 E11 and E14, and CKD by ICD-10 N18. Primary care records and estimated glomerular filtration rate (eGFR)–based definitions were not used for CKD ascertainment.

First component disease was defined as the first occurrence of any one of CVD, T2D, or CKD during follow-up. The event date was the date of the first qualifying diagnosis. Double CRMM was defined as the first occurrence of any two of the three conditions, with the event date assigned as the second qualifying diagnosis. For the specific pairwise outcomes, the event date was likewise the second qualifying diagnosis, and for triple CRMM, it was the third qualifying diagnosis.

### Covariates and mediators

Analyses were adjusted for sociodemographic characteristics, family history, genetic susceptibility, and lifestyle. Sociodemographic factors included age, sex, ethnicity (white vs non-white), educational attainment, and the Townsend Deprivation Index (TDI). Education was categorized by highest qualification: high (college/university degree), intermediate (A/AS levels or O levels/GCSEs or equivalent), and low (no qualification). The TDI, derived from census output areas, was categorized into tertiles (higher values indicate greater deprivation). Family history of CVD and T2D was self-reported by participants for first-degree relatives (biological parents or siblings). The BMI-polygenic risk score (PRS) was sourced from the UK Biobank PRS Release (Standard PRS, Category 301) and grouped into tertiles, with higher categories indicating a greater genetic predisposition to higher BMI or obesity.

Lifestyle was summarized using the Life’s Essential 4 (LE4) score derived from four LE8 domains: diet, smoking, physical activity, and sleep. Diet quality was quantified with a Dietary Approaches to Stop Hypertension (DASH)-based index from a 24-hour dietary recall; baseline touchscreen questionnaires captured frequency and duration of moderate- and vigorous-intensity physical activity, habitual sleep duration, and nicotine exposure. According to the American Heart Association (AHA) scoring algorithm, each component was scaled from 0 to 100, and the LE4 score was calculated as the average of the non-missing component scores, if at least one component was available (details in Table S1) [19]. To facilitate comparability across participant groups and ensure adequate sample size within joint trajectory-lifestyle categories, we dichotomized the LE4 score into high and low categories using the cohort-specific median as the cut point.

Chronic inflammatory-metabolic burden was quantified using the CTI. At baseline, venous samples were analyzed centrally (Beckman Coulter AU5800) to obtain high-sensitivity C-reactive protein (hs-CRP, mg/L), triglycerides (TG, mg/dL), and glucose (mg/dL). The TyG index was calculated as ln [TG (mg/dL) × glucose (mg/dL)/2]. The CTI was then computed as 0.412 × ln [hs-CRP (mg/L)] + TyG. In all causal mediation analyses, CTI was modeled as a continuous mediator, with higher values reflecting greater inflammatory-metabolic load.

### Statistical analyses

We summarized baseline characteristics for each of the nine childhood-to-adulthood body size trajectories. Continuous variables were reported as means and standard deviations, and categorical variables as counts and percentages. Incidence rates for main outcomes were expressed per 1,000 person-years. Missing covariate values were imputed using a single-imputation strategy: mean imputation for continuous variables and a missing-indicator category for categorical variables.

Time-to-event associations were estimated with Cox proportional hazards models using time since baseline as the time scale, yielding hazard ratios (HRs) and 95% confidence intervals (CIs). Proportional hazards were checked with Schoenfeld residuals and no material violations were detected. Models were adjusted sequentially: Model 1 included age and sex; Model 2 additionally included ethnicity, educational attainment, and TDI; Model 3 further adjusted for family history of CVD and T2D, the BMI-PRS, and LE4. BMI-PRS was included to account for underlying genetic susceptibility to adiposity and to partially control for shared genetic influences on body size trajectories and cardiometabolic outcomes. For body size trajectories, the reference category was Average-to-normal, and for lifestyle analyses, the reference was LE4-low. To examine effect modification, interaction terms for trajectory × LE4 were included, and HRs were reported for the joint trajectory-LE4 categories.

Causal mediation analyses were conducted to evaluate CTI as a potential mediator, using the *regmedint* package (version 1.0.1) under the counterfactual framework. For multi-category exposures, binary contrasts were constructed by comparing each adverse trajectory with the reference group (Average-to-normal). Path *a* was modeled with linear regression and path *b* with Cox regression, both adjusted for covariates included in Model 3. We estimated the average causal mediation effect (ACME), average direct effect (ADE), total effect, and the proportion mediated.

Multi-state models, which can be viewed as an extension of competing risks models, have been increasingly used to study the progression of complex diseases [26]. To characterize disease progression, we fitted two complementary multi-state Cox models with a clock-reset time scale, allowing each transition to have its own baseline hazard. Analyses were implemented in the *mstate* package (version 0.3.2) in R. The comorbidity-count model comprised the states: healthy, first component disease, double CRMM, triple CRMM, and death. Death was treated as an absorbing state, thereby accounting for competing risks between disease progression and mortality. When death occurred on the same calendar date as a diagnosis, the diagnosis time was set 0.5 days earlier to avoid ties. Participants who were diagnosed with two or more component diseases on the same date were excluded because the temporal sequence of state transitions could not be determined in the multi-state model. The comorbidity-pattern model included the states healthy, CVD, T2D, CKD, the three pairwise combinations (T2D-CVD, CVD-CKD, T2D-CKD) and triple CRMM. Transitions were modeled from healthy to each single disease, from single to double combinations, and from double to triple CRMM. We used the Aalen-Johansen estimator to obtain transition probabilities and state-occupation probabilities and derived the ELOS as the inverse of the sum of outgoing hazards. Transition probabilities and ELOS were summarized at 10 and 15 years using interpolation and trapezoidal integration. For each transition, multi-state Cox models estimated HRs, adjusting for the same covariates as Model 3.

Prespecified subgroup analyses evaluated potential heterogeneity by sex and age (<60 vs. ≥60 years). Robustness of the findings was further assessed in sensitivity analyses that (i) excluded events occurring within the first two years of follow-up, (ii) repeated analyses using complete cases for covariates, (iii) excluded participants with underweight (BMI <18.5 kg/m^2^), (iv) addressed missing covariate data using multiple imputation by chained equations (MICE), generating five imputed datasets in which the fully adjusted models were refitted and pooled using Rubin’s rules, and (v) conducted analyses in which Model 3 was re-estimated without adjustment for BMI-PRS. All statistical tests were two-sided, and a *P* value <0.05 was considered statistically significant. Analyses were conducted in R version 4.2.2 (R Foundation for Statistical Computing, Vienna, Austria).

## Results

### Descriptive results

A total of 398,491 participants were included in the main analyses (Figure S1). Baseline characteristics across the nine body size trajectory groups are summarized in Table S2. Significant differences were observed across groups in demographics, socioeconomic status, genetic risk, lifestyle behaviors, and CTI (all *P* < 0.05). Participants with sustained or progressive obesity (e.g., Thinner-to-Obesity, Plumper-to-Obesity) were more likely to be male, socioeconomically disadvantaged, and exhibit poorer lifestyle and metabolic profiles, including elevated CTI. Over a median follow-up of 13.5 years (interquartile range (IQR): 12.6–14.4), 76,500 participants (19.24%) developed single CRMM and 33,786 (8.50%) participants died. As shown in Table 1, the incidence rate was highest for first component disease and lowest for triple CRMM, with all-cause mortality occurring at a moderate rate.

**Table 1:** Associations of childhood-to-adulthood body size trajectories with disease incidence and mortality outcomes (N = 398,491)

	**First component disease** **HR (95% CI)**	**Double CRMM** **HR (95% CI)**	**T2D-CVD** **HR (95% CI)**	**CVD-CKD** **HR (95% CI)**	**T2D-CKD** **HR (95% CI)**	**Triple CRMM** **HR (95% CI)**	**All-cause mortality** **HR (95% CI)**
**Cases/1000 PYs**	15.53 (15.42, 15.64)	2.68 (2.64, 2.73)	1.50 (1.47, 1.53)	1.07 (1.04, 1.10)	0.76 (0.74, 0.78)	0.33 (0.32, 0.35)	6.32 (6.26, 6.39)
**Model 1**							
**Body size trajectories**							
Thinner-to-normal	1.10 (1.07, 1.14)	1.19 (1.08, 1.31)	1.36 (1.17, 1.57)	1.06 (0.93, 1.21)	1.39 (1.13, 1.72)	1.35 (0.97, 1.89)	0.98 (0.94, 1.02)
Thinner-to-overweight	1.72 (1.67, 1.78)	2.46 (2.27, 2.66)	3.53 (3.13, 3.99)	1.70 (1.52, 1.90)	3.19 (2.68, 3.81)	3.38 (2.57, 4.45)	0.94 (0.91, 0.98)
Thinner-to-obesity	3.13 (3.03, 3.23)	6.21 (5.73, 6.72)	9.61 (8.52, 10.85)	3.40 (3.03, 3.82)	11.48 (9.70, 13.59)	11.72 (8.99, 15.27)	1.33 (1.27, 1.40)
Average-to-normal	Ref	Ref	Ref	Ref	Ref	Ref	Ref
Average-to-overweight	1.44 (1.40, 1.48)	1.88 (1.74, 2.03)	2.39 (2.12, 2.70)	1.56 (1.40, 1.73)	2.27 (1.91, 2.69)	2.50 (1.91, 3.27)	0.93 (0.90, 0.97)
Average-to-obesity	2.59 (2.51, 2.66)	4.74 (4.40, 5.12)	7.29 (6.49, 8.20)	2.87 (2.58, 3.19)	8.23 (6.98, 9.70)	9.00 (6.95, 11.65)	1.17 (1.13, 1.22)
Plumper-to-normal	0.98 (0.92, 1.04)	0.88 (0.73, 1.06)	0.97 (0.72, 1.30)	0.78 (0.60, 1.03)	1.01 (0.67, 1.52)	0.87 (0.43, 1.75)	1.15 (1.07, 1.23)
Plumper-to-overweight	1.51 (1.46, 1.57)	2.05 (1.85, 2.27)	2.55 (2.19, 2.97)	1.65 (1.43, 1.90)	2.77 (2.24, 3.42)	2.72 (1.95, 3.81)	1.05 (1.00, 1.10)
Plumper-to-obesity	2.82 (2.73, 2.91)	5.17 (4.76, 5.62)	8.04 (7.10, 9.10)	2.90 (2.56, 3.28)	9.27 (7.79, 11.03)	9.68 (7.37, 12.72)	1.33 (1.27, 1.40)
**Model 2**							
**Body size trajectories**							
Thinner-to-normal	1.10 (1.06, 1.14)	1.19 (1.08, 1.31)	1.35 (1.16, 1.57)	1.06 (0.93, 1.21)	1.38 (1.12, 1.70)	1.34 (0.96, 1.87)	0.98 (0.94, 1.02)
Thinner-to-overweight	1.66 (1.61, 1.71)	2.31 (2.14, 2.51)	3.33 (2.95, 3.76)	1.61 (1.44, 1.80)	2.97 (2.49, 3.54)	3.15 (2.39, 4.15)	0.91 (0.88, 0.95)
Thinner-to-obesity	2.85 (2.76, 2.94)	5.34 (4.93, 5.79)	8.22 (7.28, 9.28)	2.96 (2.63, 3.32)	9.67 (8.16, 11.45)	9.79 (7.50, 12.77)	1.22 (1.16, 1.27)
Average-to-normal	Ref	Ref	Ref	Ref	Ref	Ref	Ref
Average-to-overweight	1.41 (1.38, 1.45)	1.83 (1.70, 1.98)	2.34 (2.08, 2.64)	1.51 (1.36, 1.68)	2.20 (1.85, 2.61)	2.43 (1.86, 3.18)	0.92 (0.89, 0.95)
Average-to-obesity	2.46 (2.39, 2.53)	4.38 (4.05, 4.72)	6.74 (6.00, 7.58)	2.64 (2.37, 2.94)	7.51 (6.37, 8.86)	8.17 (6.31, 10.58)	1.11 (1.07, 1.16)
Plumper-to-normal	0.99 (0.93, 1.05)	0.89 (0.74, 1.08)	0.98 (0.73, 1.32)	0.79 (0.61, 1.03)	1.02 (0.68, 1.54)	0.88 (0.44, 1.77)	1.15 (1.07, 1.23)
Plumper-to-overweight	1.49 (1.43, 1.55)	2.00 (1.81, 2.21)	2.49 (2.14, 2.90)	1.60 (1.39, 1.85)	2.68 (2.17, 3.31)	2.64 (1.89, 3.70)	1.03 (0.98, 1.08)
Plumper-to-obesity	2.70 (2.61, 2.79)	4.80 (4.41, 5.21)	7.47 (6.60, 8.46)	2.67 (2.36, 3.03)	8.49 (7.14, 10.11)	8.80 (6.70, 11.57)	1.25 (1.20, 1.32)
**Model 3**							
**Body size trajectories**							
Thinner-to-normal	1.10 (1.06, 1.13)	1.17 (1.07, 1.29)	1.33 (1.15, 1.55)	1.06 (0.93, 1.21)	1.37 (1.11, 1.69)	1.32 (0.95, 1.85)	0.98 (0.94, 1.02)
Thinner-to-overweight	1.61 (1.57, 1.66)	2.22 (2.05, 2.40)	3.14 (2.78, 3.55)	1.58 (1.41, 1.77)	2.84 (2.38, 3.39)	2.99 (2.27, 3.93)	0.91 (0.87, 0.95)
Thinner-to-obesity	2.70 (2.62, 2.79)	4.95 (4.57, 5.37)	7.44 (6.59, 8.41)	2.85 (2.53, 3.20)	8.91 (7.52, 10.56)	8.91 (6.82, 11.63)	1.19 (1.13, 1.25)
Average-to-normal	Ref	Ref	Ref	Ref	Ref	Ref	Ref
Average-to-overweight	1.39 (1.35, 1.43)	1.78 (1.65, 1.93)	2.26 (2.00, 2.55)	1.49 (1.34, 1.66)	2.13 (1.80, 2.54)	2.35 (1.79, 3.07)	0.91 (0.88, 0.95)
Average-to-obesity	2.35 (2.28, 2.42)	4.09 (3.79, 4.42)	6.17 (5.48, 6.94)	2.55 (2.29, 2.84)	6.96 (5.90, 8.22)	7.51 (5.79, 9.73)	1.09 (1.04, 1.13)
Plumper-to-normal	0.96 (0.91, 1.02)	0.86 (0.71, 1.04)	0.94 (0.70, 1.26)	0.77 (0.59, 1.01)	0.99 (0.66, 1.48)	0.84 (0.42, 1.69)	1.13 (1.05, 1.21)
Plumper-to-overweight	1.43 (1.38, 1.49)	1.90 (1.71, 2.10)	2.33 (2.00, 2.71)	1.56 (1.35, 1.79)	2.53 (2.05, 3.14)	2.47 (1.77, 3.46)	1.01 (0.96, 1.06)
Plumper-to-obesity	2.53 (2.45, 2.62)	4.38 (4.03, 4.77)	6.63 (5.85, 7.52)	2.56 (2.26, 2.90)	7.71 (6.47, 9.19)	7.89 (5.99, 10.39)	1.22 (1.16, 1.28)

Model 1 was adjusted for age, sex. Model 2 was adjusted for model 1+ educational attainment, ethnicity and Townsend Deprivation Index. Model 3 was adjusted for model 2+ family history of CVD, family history of T2D, polygenic risk score for BMI, and LE4 score groups. LE4 indicates 4 lifestyle metrics from Life’s Essential 8. First component disease denotes the first occurrence of any one of CVD, T2D, or CKD during follow-up. Double CRMM denotes the first attainment of any two of these conditions (i.e., T2D-CVD, CVD-CKD, or T2D-CKD); the event date is the date of the second qualifying diagnosis. Triple CRMM denotes the occurrence of all three conditions; the event date is the date of the third qualifying diagnosis. Abbreviations: CRMM, cardio-renal-metabolic multimorbidity; CVD, cardiovascular disease; T2D, type 2 diabetes; CKD, chronic kidney disease; HR, hazard ratio; CI, confidence interval; PYs, person-years; Ref, reference.

### Associations of body size trajectories with disease incidence and mortality

In the fully adjusted model (Model 3, Table 1), compared with the reference group (Average-to-normal), body size trajectories showed a clear risk gradient. HRs for first, double, and triple CRMM increased progressively from Average-to-obesity (2.35, 4.09, and 7.51) to Plumper-to-obesity (2.53, 4.38, and 7.89) and Thinner-to-obesity (2.70, 4.95, and 8.91). Corresponding HRs for all-cause mortality were 1.09, 1.22, and 1.19, respectively. In contrast, the Plumper-to-normal trajectory was essentially neutral for CRMM risk but showed a modestly higher mortality rate.

### Combined risk stratification by body size trajectory and LE4 score

Using the Average-to-normal/LE4-high group as the reference, a clear risk gradient was observed across cross-classified categories (Table 2). Participants with adverse trajectories (Thinner-to-obesity or Plumper-to-obesity) and low LE4 scores had the highest risk, those with favorable trajectories (Thinner-to-normal or Plumper-to-normal) and high LE4 scores had the lowest risk; other groups exhibited intermediate risks. The pattern was consistent across first-component, double, and triple CRMM as well as all-cause mortality. Within the same trajectory stratum, moving from LE4-low to LE4-high partially attenuated risks. For Average-to-obesity, HRs decreased from 2.81 to 2.43 for first-component (−21.0%), 5.31 to 4.14 for double (−27.1%), and 10.38 to 7.87 for triple CRMM (−26.8%). Corresponding reductions were observed for Plumper-to-obesity (from 3.15 to 2.49, −30.7%; from 5.92 to 4.18, −35.4%; from 11.25 to 7.91, −32.6%) and Thinner-to-obesity (from 3.21 to 2.83, −17.2%; from 6.44 to 5.00, −26.5%; from 13.10 to 8.53, −37.8%). All-cause mortality showed a similar attenuation pattern.

**Table 2:** Hazard ratios (95% CIs) for first-component, double, and triple cardio-renal-metabolic multimorbidity (CRMM) and all-cause mortality by body size trajectories × LE4 score groups (N = 398,491)

	**First component disease** **HR (95% CI)**	**Double CRMM** **HR (95% CI)**	**T2D-CVD** **HR (95% CI)**	**CVD-CKD** **HR (95% CI)**	**T2D-CKD** **HR (95% CI)**	**Triple CRMM** **HR (95% CI)**	**All-cause mortality** **HR (95% CI)**
**Body size trajectory/LE4 group**							
Thinner-to-normal/LE4-low	1.33 (1.27, 1.39)	1.56 (1.37, 1.79)	2.02 (1.64, 2.49)	1.36 (1.13, 1.64)	1.47 (1.09, 1.99)	1.64 (1.00, 2.67)	1.49 (1.41, 1.59)
Thinner-to-normal/LE4-high	1.11 (1.06, 1.17)	1.15 (1.00, 1.33)	1.23 (0.98, 1.55)	1.12 (0.93, 1.36)	1.43 (1.06, 1.91)	1.55 (0.96, 2.51)	1.04 (0.98, 1.11)
Thinner-to-overweight/LE4-low	1.94 (1.86, 2.02)	2.83 (2.52, 3.17)	4.23 (3.54, 5.07)	1.98 (1.69, 2.32)	3.23 (2.53, 4.12)	3.63 (2.42, 5.44)	1.31 (1.23, 1.39)
Thinner-to-overweight/LE4-high	1.66 (1.59, 1.73)	2.29 (2.04, 2.57)	3.40 (2.83, 4.09)	1.73 (1.47, 2.03)	2.81 (2.20, 3.60)	3.57 (2.39, 5.33)	1.04 (0.98, 1.10)
Thinner-to-obesity/LE4-low	3.21 (3.08, 3.36)	6.44 (5.75, 7.22)	10.29 (8.61, 12.30)	3.90 (3.32, 4.59)	10.82 (8.57, 13.67)	13.10 (8.91, 19.26)	1.67 (1.56, 1.78)
Thinner-to-obesity/LE4-high	2.83 (2.70, 2.96)	5.00 (4.44, 5.63)	7.84 (6.52, 9.43)	2.80 (2.35, 3.33)	8.19 (6.44, 10.41)	8.53 (5.71, 12.72)	1.41 (1.31, 1.51)
Average-to-normal/LE4-low	1.23 (1.18, 1.29)	1.31 (1.14, 1.50)	1.44 (1.16, 1.79)	1.36 (1.14, 1.62)	1.12 (0.82, 1.52)	1.43 (0.88, 2.33)	1.59 (1.51, 1.69)
Average-to-normal/LE4-high	Ref	Ref	Ref	Ref	Ref	Ref	Ref
Average-to-overweight/LE4-low	1.68 (1.61, 1.75)	2.40 (2.15, 2.68)	3.15 (2.63, 3.76)	2.13 (1.84, 2.48)	2.80 (2.21, 3.55)	3.65 (2.47, 5.39)	1.33 (1.26, 1.40)
Average-to-overweight/LE4-high	1.42 (1.37, 1.48)	1.72 (1.54, 1.93)	2.35 (1.96, 2.82)	1.40 (1.20, 1.64)	1.78 (1.39, 2.28)	2.11 (1.41, 3.15)	1.02 (0.97, 1.08)
Average-to-obesity/LE4-low	2.81 (2.70, 2.93)	5.31 (4.76, 5.92)	8.46 (7.11, 10.08)	3.32 (2.84, 3.86)	8.50 (6.76, 10.68)	10.38 (7.11, 15.17)	1.55 (1.46, 1.64)
Average-to-obesity/LE4-high	2.43 (2.33, 2.54)	4.14 (3.70, 4.64)	6.56 (5.49, 7.84)	2.70 (2.31, 3.16)	6.36 (5.04, 8.03)	7.87 (5.35, 11.56)	1.26 (1.19, 1.34)
Plumper-to-normal/LE4-low	1.26 (1.16, 1.37)	1.44 (1.14, 1.81)	1.63 (1.13, 2.33)	1.33 (0.96, 1.84)	1.65 (1.03, 2.67)	1.60 (0.70, 3.65)	1.72 (1.56, 1.89)
Plumper-to-normal/LE4-high	0.89 (0.81, 0.98)	0.55 (0.38, 0.78)	0.64 (0.37, 1.11)	0.50 (0.31, 0.82)	0.46 (0.20, 1.06)	0.45 (0.11, 1.87)	1.20 (1.08, 1.34)
Plumper-to-overweight/LE4-low	1.74 (1.65, 1.84)	2.37 (2.06, 2.73)	3.30 (2.67, 4.10)	1.91 (1.56, 2.33)	2.95 (2.20, 3.95)	3.58 (2.23, 5.74)	1.49 (1.38, 1.60)
Plumper-to-overweight/LE4-high	1.45 (1.37, 1.54)	2.01 (1.73, 2.32)	2.37 (1.87, 2.99)	1.75 (1.43, 2.15)	2.46 (1.81, 3.33)	2.44 (1.46, 4.06)	1.12 (1.03, 1.21)
Plumper-to-obesity/LE4-low	3.15 (3.01, 3.30)	5.92 (5.26, 6.65)	9.37 (7.81, 11.24)	3.51 (2.96, 4.17)	9.73 (7.66, 12.35)	11.25 (7.57, 16.71)	1.79 (1.67, 1.91)
Plumper-to-obesity/LE4-high	2.49 (2.37, 2.62)	4.18 (3.69, 4.74)	6.75 (5.57, 8.18)	2.51 (2.08, 3.03)	6.71 (5.21, 8.63)	7.91 (5.23, 11.96)	1.36 (1.26, 1.46)

Models were adjusted for age, sex, educational attainment, ethnicity and Townsend Deprivation Index, family history of CVD, family history of T2D, polygenic risk score for BMI and LE4 score. LE4 indicates 4 lifestyle metrics from Life’s Essential 8. First component disease denotes the first occurrence of any one of CVD, T2D, or CKD during follow-up. Double CRMM denotes the first attainment of any two of these conditions (i.e., T2D-CVD, CVD-CKD, or T2D-CKD); the event date is the date of the second qualifying diagnosis. Triple CRMM denotes the occurrence of all three conditions; the event date is the date of the third qualifying diagnosis. Abbreviations: CRMM, cardio-renal-metabolic multimorbidity; CVD, cardiovascular disease; T2D, type 2 diabetes; CKD, chronic kidney disease; HR, hazard ratio; CI, confidence interval; Ref, reference.

### Mediation by CTI in associations of body size trajectories with CRMM outcomes

**Fig. 2:**
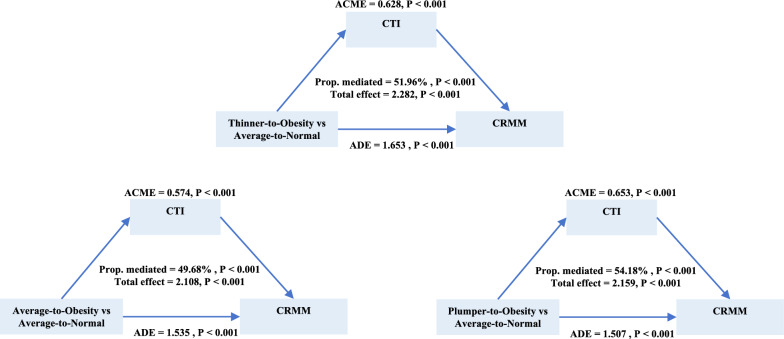
Mediation effects of CTI on the associations between childhood-to-adulthood body size trajectories and the onset of CRMM. The figure shows the estimated total effects, ADE, and ACME of CTI on the associations of childhood-to-adulthood body size trajectories (Thinner-to-Obesity, Average-to-Obesity, and Plumper-to-Obesity vs Average-to-Normal) with CRMM. Proportion mediated and corresponding *P* values are presented. All models were adjusted for age, sex, educational attainment, ethnicity and Townsend Deprivation Index, family history of CVD, family history of T2D, LE4 score and polygenic risk score for BMI. LE4 indicates 4 lifestyle metrics from Life’s Essential 8. Abbreviations: CTI, C-reactive protein-triglyceride glucose index; CVD, cardiovascular disease; T2D, type 2 diabetes; CKD, chronic kidney disease; CRMM, cardio-renal-metabolic multimorbidity; ADE, average direct effect; ACME, average causal mediation effect

After full adjustment, CTI significantly mediated the associations of adverse trajectories (Thinner-to-Obesity, Average-to-Obesity, and Plumper-to-Obesity) and CRMM outcomes compared with the Average-to-Normal group (Fig. 2; Figure S2). Across outcomes and trajectories, the proportion mediated ranged from approximately 42% to 54%, indicating that nearly half of the excess risk associated with sustained or progressive obesity is attributable to inflammatory-metabolic pathways captured by CTI.

### Multi-state transitions along the comorbidity-count progression pathway across body size trajectories

**Fig. 3:**
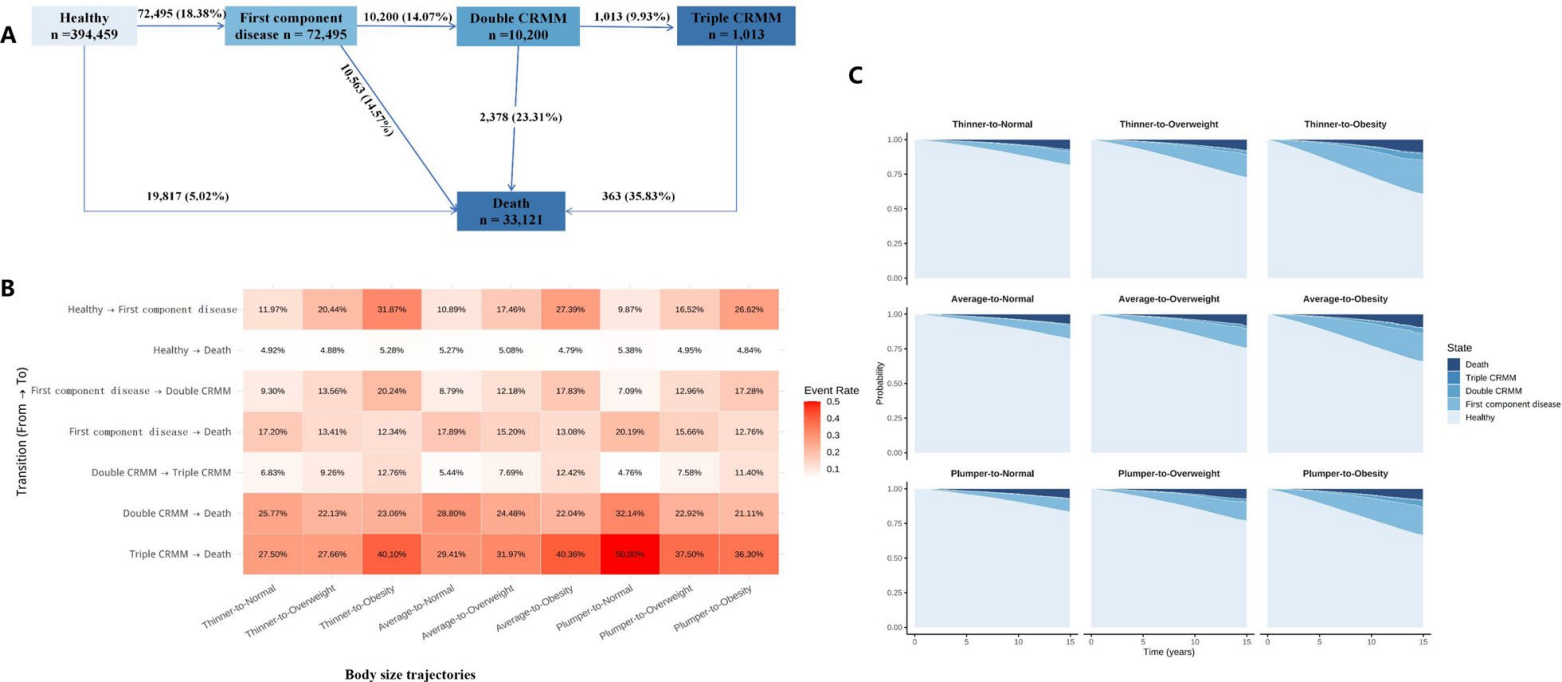
Multi-state model estimates of transitions along the comorbidity-count progression pathway across childhood-to-adulthood body size trajectories (N = 394,459). (**A**) Multi-state transition diagram illustrating progression from healthy status to first component disease, from first component disease to double CRMM, from double CRMM to triple CRMM, and from each state to death. (**B**) Transition probability heatmap (%) showing the proportion of individuals transitioning from one health state to another across childhood-to-adulthood body size trajectory groups. Darker colors indicate higher transition probabilities. (**C**) Stacked state occupation probability plots displaying the proportion of individuals in each health state over 15 years of follow-up by childhood-to-adulthood body size trajectory groups, reflecting the temporal dynamics of disease progression and the timing of state transitions

After excluding 4,032 participants with simultaneous diagnoses of multiple component diseases, 394,459 participants were included in the multi-state analyses (Figure S1). Figure. 3A illustrates multi-state transitions from healthy to first, double, and triple CRMM, and from each state to death. The largest flow occurred from healthy to first component disease and declined progressively thereafter; deaths were observed at all stages, with higher proportions following double and triple CRMM. The transition-probability heatmap (Fig. 3B) revealed a clear trajectory gradient—lower probabilities in favorable trajectories and higher probabilities in adverse ones, particularly for transitions from healthy to first component disease and from CRMM states to death. As shown in Fig. 3C, individuals with adverse trajectories exited the healthy state earlier and accumulated more time in CRMM states, consistent with ELOS patterns at 10 and 15 years (Table S3). At 15 years, the healthy-state occupation and ELOS were 82.26% and 13.08 years for Average-to-Normal, compared with 60.91% and 10.59 years for Thinner-to-Obesity. Correspondingly, time spent in CRMM states was longer for Thinner-to-Obesity than for Average-to-Normal (first component disease ELOS: 2.85 vs. 1.04 years; double CRMM: 0.49 vs. 0.12 years).

### Multi-state Cox estimates for transition risks along the comorbidity-count progression pathway across body size trajectories

**Fig. 4:**
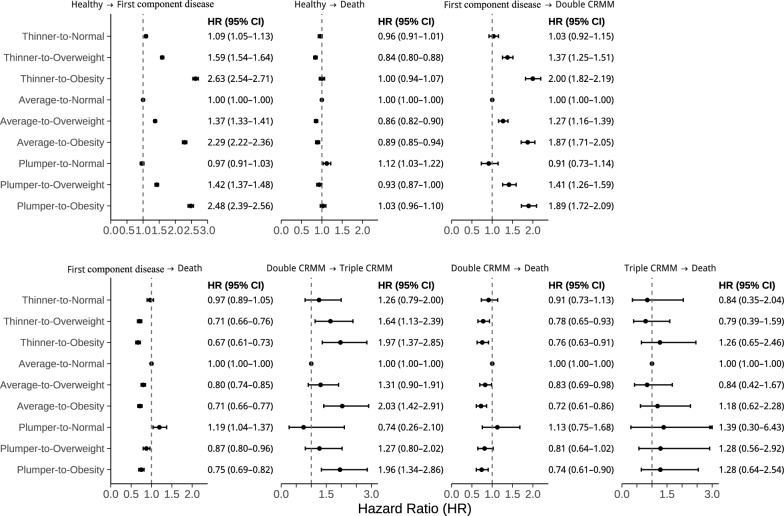
Associations of childhood-to-adulthood body size trajectories with transitions along the comorbidity-count progression pathway (Healthy → First component disease → Double CRMM → Triple CRMM → Death) in multi-state models (N = 394,459). Models were adjusted for age, sex, educational attainment, ethnicity and Townsend Deprivation Index, family history of cardiovascular diseases, family history of type 2 diabetes, LE4 score and polygenic risk score for BMI. LE4 indicates 4 lifestyle metrics from Life’s Essential 8. Abbreviation: CRMM, cardio-renal-metabolic multimorbidity

Figure 4 summarizes transition-specific HRs from the multi-state Cox models along the comorbidity-count pathway. Using Average-to-Normal as the reference, HRs increased stepwise from favorable to adverse trajectories, with the steepest gradients observed for transitions from healthy to first component disease. For example, for the healthy-to-first component disease transition, HRs were 1.37 for the Average-to-Overweight, 2.29 for Average-to-Obesity, 2.48 for Plumper-to-Obesity, and 2.63 for Thinner-to-Obesity.

### Multi-state transitions along the comorbidity-pattern progression pathway across body size trajectories

**Fig. 5:**
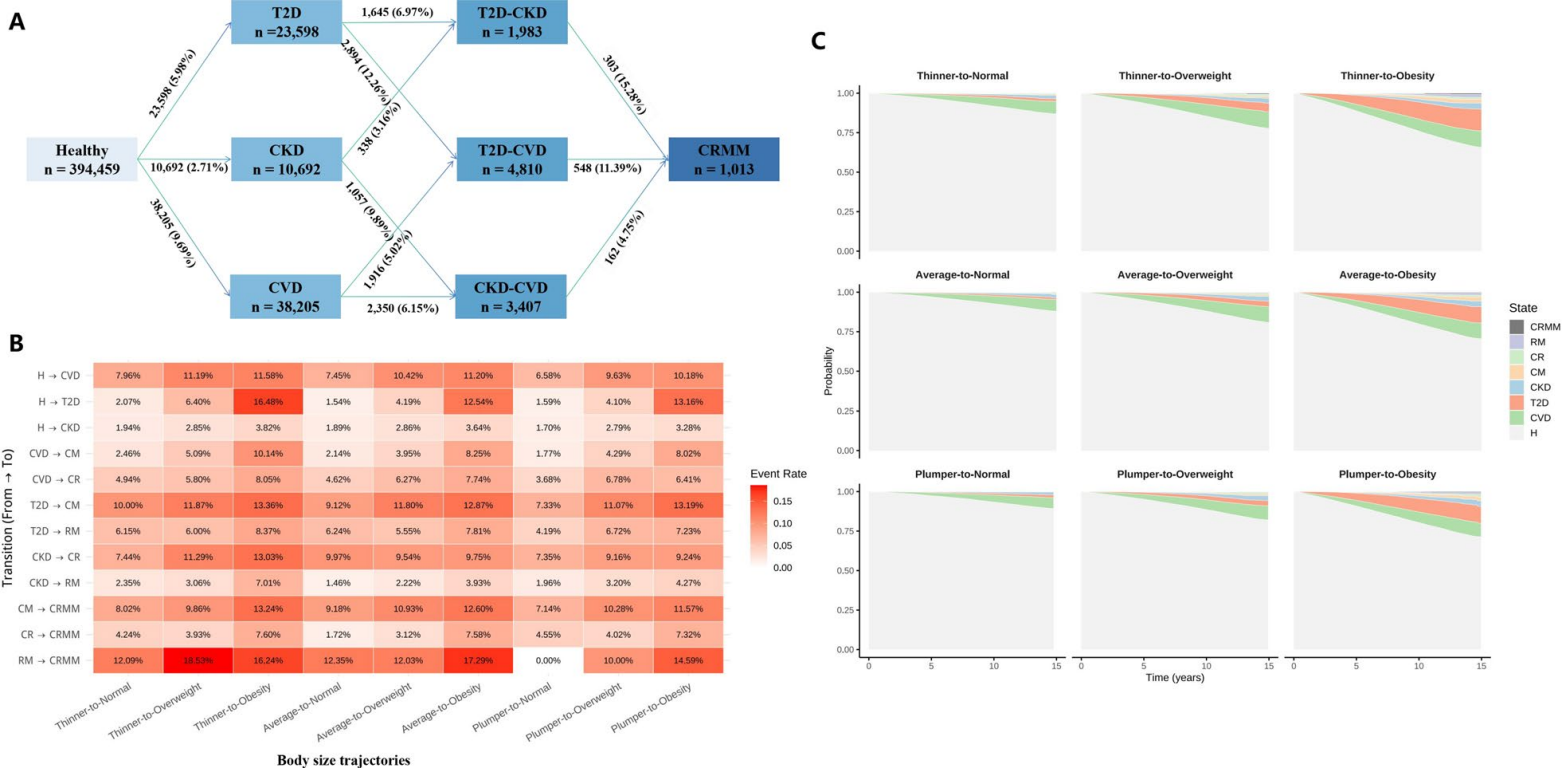
Multi-state model estimates of transitions along the comorbidity-pattern progression pathway across childhood-to-adulthood body size trajectories (N = 394,459). (**A**) Multi-state transition diagram illustrating progression from healthy status to different comorbidity-patterns of CRMM and then to triple CRMM. (**B**) Transition probability heatmap (%) showing the proportion of individuals transitioning from one health state to another across childhood-to-adulthood body size trajectory groups. Darker colors indicate higher transition probabilities. (**C**) Stacked state occupation probability plots displaying the proportion of individuals in each health state over 15 years of follow-up by childhood-to-adulthood body size trajectory groups, reflecting the temporal dynamics of disease progression and the timing of state transitions. Abbreviation: CVD, cardiovascular disease; T2D, type 2 diabetes; CKD, chronic kidney disease; CRMM, cardio-renal-metabolic multimorbidity

Based on observed transitions in this cohort, CVD was the most common first-diagnosed disease (Fig. 5A). Among double-comorbidity patterns, T2D-CVD occurred most frequently, whereas T2D-CKD was least frequent; however, the transition from T2D-CKD to triple CRMM had the highest proportion, suggesting that double patterns involving T2D act as key hubs for progression to triple CRMM. The transition-probability heatmap (Fig. 5B) revealed a clear gradient across body size trajectories, with lower probabilities in favorable trajectories and higher probabilities in adverse ones, particularly for transitions from the healthy state to T2D and from double to triple CRMM. Figure 5C indicates that adverse trajectories exited the healthy state earlier and accumulated more time in single- and double-disease states, with the longest duration observed in the T2D single-disease state. For example, in the Thinner-to-Obesity trajectory, healthy-state occupation at 15 years was 65.77%, with an ELOS of 1.62 years in the T2D state, considerably longer than other trajectories (Table S4). By contrast, the Average-to-Normal trajectory maintained 87.85% healthy-state occupation, with an ELOS of 0.15 years in the T2D state.

### Multi-state Cox estimates for transition risks along the comorbidity-pattern progression pathway across body size trajectories

**Fig. 6:**
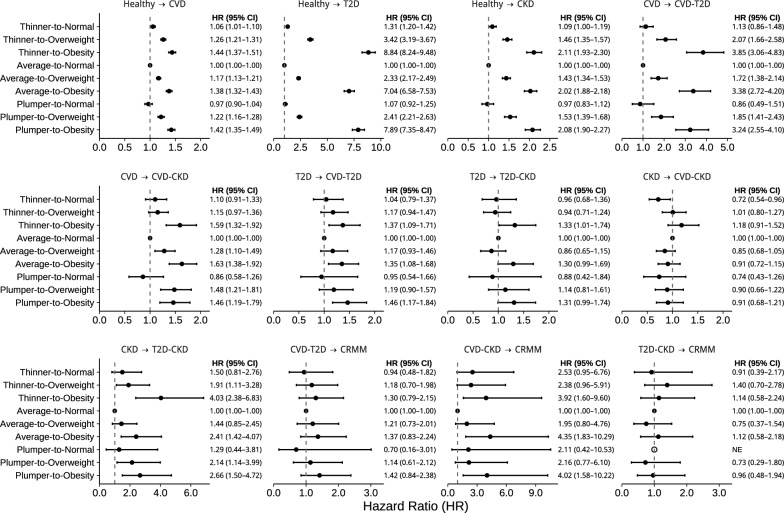
Associations of childhood-to-adulthood body size trajectories with transitions along the comorbidity-pattern progression pathway in multi-state models (N = 394,459). Models were adjusted for age, sex, educational attainment, ethnicity and Townsend Deprivation Index, family history of CVD, family history of T2D, LE4 score and polygenic risk score for BMI. LE4 indicates 4 lifestyle metrics from Life’s Essential 8. NE = not estimable due to sparse events or (quasi-)complete separation in the Cox model. Abbreviations: CVD, cardiovascular disease; T2D, type 2 diabetes; CKD, chronic kidney disease; CRMM, cardio-renal-metabolic multimorbidity

Using Average-to-Normal as the reference, entry transitions exhibited the steepest risk gradients, particularly from the healthy state to T2D. HRs were substantially higher in adverse trajectories, whereas increases for transitions to CVD or CKD were more moderate (Fig. 6). For single-to-double transitions, obesity-related trajectories showed the greatest increases when the destination included T2D: for CVD to T2D-CVD, HRs were 3.85, 3.38, and 3.24 for Thinner-, Average-, and Plumper-to-Obesity, respectively; for CKD to T2D-CKD, HRs were 4.03 for Thinner-to-Obesity and 2.66 for Plumper-to-Obesity. For double-to-triple transitions, adverse obesity trajectories conferred the highest hazards from T2D-CVD and CVD-CKD to triple CRMM. Overall, adverse body size trajectories were associated with elevated risks across most transitions, with the strongest gradients observed along T2D-related pathways.

### Additional analyses

The risk gradient across body size trajectories remained consistent across age and sex strata, with hazards increasing stepwise from favorable to adverse trajectories, most pronounced for transitions from healthy to first component disease (Figures S3-S6; Tables S5-S6). Age-stratified analyses indicated stronger effects in participants <60 years; for instance, the HR for first-component CRMM in the Thinner-to-Obesity trajectory was 3.21 in those <60 versus 2.42 in those ≥60. Sex-stratified patterns were directionally similar, with only modest differences in magnitude. Sensitivity analyses confirmed the robustness of these findings (Tables S7-S11; Figures S7-S13), as effect directions and stepwise trajectory gradients were preserved across single-disease Cox models and both multi-state models, with only minor changes in magnitude. Analyses using multiple imputation for missing covariates were consistent with the primary analyses (Table S10). Furthermore, inclusion of BMI-PRS had little impact on the observed associations (Table S11). Overall, all key inferences remained unchanged.

## Discussion

In this large, long-term cohort analyzed using unified multi-state frameworks, adverse life-course body size trajectories were associated with higher transition hazards along the comorbidity-count pathway and earlier exit from the healthy state, resulting in shorter healthy-state duration and longer occupancy of CRMM states. Pattern-based analyses further refined the progression landscape, indicating that comorbidities including T2D act as key hubs for escalation to triple CRMM. Joint stratification by trajectory and LE4 lifestyle score revealed a clear stepwise gradient from favorable/LE4-high to adverse/LE4-low, and a shift from LE4-low to LE4-high consistently attenuated the excess risk. Mediation analyses suggested that low-grade inflammation and insulin resistance, captured by the composite CTI, explained approximately half of the association between trajectories and outcomes.

Within a multi-state framework, we characterized the overall features of CRMM progression in terms of both comorbidity counts and disease-pattern transitions, and we quantified transition probabilities, state-occupation probabilities, and expected length of stay. Transitions were steepest when the destination state included T2D, and dyads that include T2D (e.g., T2D-CKD, T2D-CVD) served as key hubs for escalation to triple disease. This pattern was consistent with longitudinal studies based on the UK Biobank [20]. Similar findings have been observed in a multi-state analysis of U.S. Medicare beneficiaries aged ≥65 years, where the state space comprised death and all diagnosis combinations of three chronic conditions: DM+IHD was more likely to progress to triple disease rather than directly to death, whereas IHD+CKD was more prone to transition to death, suggesting that double-disease combinations including T2D have greater escalation potential [27]. Compared with studies that only describe cross-sectional overlap, our dynamic evidence adds essential temporal information on when transitions occur and along which pathways escalation proceeds [1]. From an integrative biological perspective, the AHA’s CKM (cardio-kidney-metabolic) framework emphasizes the coupling and shared pathways among cardiovascular, renal, and metabolic dysfunction, providing mechanistic plausibility for the observed T2D-centered hubs and accelerated progression [28]. The CKM cascade begins with early-life exposures leading to excess and dysfunctional adiposity, triggering inflammation and insulin resistance. These processes drive metabolic risk factors and CKD in parallel and, over time, converge into subclinical atherosclerosis, myocardial abnormalities, and progressive renal decline, ultimately elevating the risks of CVD and death [6]. T2D links upstream adiposity dysfunction to downstream cardio-renal injury and speeds the shift from single to double and triple disease, serving as a central hub consistent with independent evidence of its high multimorbidity burden and network centrality [29, 30].

In keeping with the integrated CKM concept, we observed that adverse body size trajectories, especially Thinner-to-Obesity, accelerate longitudinal CRMM progression, characterized by earlier exit from health, faster entry into T2D, and more frequent escalation through T2D-containing disease pairs to triple disease, with shorter healthy time and longer time in multimorbid states. This pattern aligns with the AHA CKM framework, which emphasizes that excess and dysfunctional adiposity contributes to cardio-renal injury via insulin resistance and low-grade inflammation, thereby elevating CVD and kidney risks [6, 31]. From a life-course perspective, increasing-BMI or sustained-obesity trajectories substantially raise the risk of adult-onset T2D, underscoring the importance of early weight control [32]. A UK Biobank-based study reported similar patterns: child-to-adult trajectories with increasing BMI or sustained obesity were associated with substantially higher risk of incident T2D, with the highest risk among those who were thinner in childhood but obese in adulthood [14]. Regarding overall multimorbidity burden, a large multicohort study shows that obesity markedly increases the risks of simple and complex multimorbidity and reduces disease-free survival [33].

Extending these results, we observed consistent risk attenuation within the same body size trajectory when moving from LE4-low to LE4-high, particularly in the Average-to-Obesity, Plumper-to-Obesity, and Thinner-to-Obesity groups. These within-trajectory gains align with UK Biobank multi-state evidence that a higher composite lifestyle score is protective across all CRMM transitions [20]. Complementary multi-state studies focused on T2D patients have shown that healthier pre-onset and post-onset lifestyles are associated with lower risks of progressing to complications and death [34]. Taken together, these observations suggest that lifestyle improvement yields meaningful risk reduction even among individuals on obesity-related trajectories, particularly in the reduction of CRMM incidence, and that early lifestyle interventions are particularly important.

In mediation analyses, CTI accounted for approximately 42% to 54% of the association between adverse body size trajectories and CRMM outcomes, suggesting that a substantial proportion of the excess risk may operate through inflammatory and insulin-resistance pathways captured by this composite marker. The signal is congruent with the prominence of T2D-related routes in our multi-state analyses, since CTI integrates hs-CRP and TyG to summarize low-grade inflammation and metabolic load that are closely aligned with glycemic worsening and downstream cardio-renal injury. Representative evidence supports this pathway as CTI is associated with higher risks of diabetes, CVD events, and mortality, including recent analyses in cohort settings [35, 36]. Recent studies show that TyG-derived metrics capture inflammatory and metabolic burden relevant to CRMM. TyG-BMI predicts adverse CRMM trajectories in multi-state models, and TyG-related indices independently associate with all-cause and cardiovascular mortality across CKM stages 0 to 3, supporting the use of CTI for pathway-level risk quantification [37, 38]. However, because this study is observational, the mediation findings should be interpreted as indicating potential pathways rather than establishing causal mechanisms. Taken together, these findings suggest that inflammation and insulin resistance may constitute important biological pathways linking body size trajectories to CRMM progression.

These findings also have important clinical and preventive implications. Because T2D-centered disease combinations appear to function as key hubs in CRMM progression, interventions targeting metabolic dysfunction may help disrupt these escalation pathways. Early weight management and lifestyle interventions that improve insulin sensitivity may delay the transition from single cardiometabolic disease to multimorbidity, consistent with evidence that lifestyle modification reduces inflammatory and metabolic burden [39]. In addition, pharmacological strategies widely used in T2D management, such as metformin and newer glucose-lowering agents with established cardio-renal protective effects, may help mitigate downstream cardiovascular and renal complications [40, 41]. Given the mediating role of inflammatory-metabolic pathways observed in this study, therapeutic strategies targeting systemic inflammation may also represent a potential approach to slowing CRMM progression.

This study, drawing on a large, long-term prospective cohort, applied a multi-state, clock-reset modeling strategy to delineate comorbidity dynamics from two complementary disease-progression trajectories: (1) a comorbidity count-escalation trajectory and (2) a composition/pattern-evolution trajectory. Within a unified framework, we estimated state-specific hazards, transition probabilities, state-occupation probabilities, and expected length of stay, thereby clarifying the temporal ordering and pace of comorbidity emergence and worsening while enhancing statistical power and inferential robustness.

Several limitations should be acknowledged. First, the UK Biobank exhibits a healthy-volunteer profile—participants tend to smoke less, drink less frequently, and have lower BMI than the general population—potentially limiting external validity and absolute risk estimates; nonetheless, relative associations with mortality generally mirror those observed in more representative national cohorts [42]. Second, childhood body size was obtained retrospectively and is therefore vulnerable to recall error and potential exposure misclassification, although prior studies report reasonable concordance with measured childhood BMI [43, 44]. Because this misclassification is unlikely to be related to subsequent disease outcomes, it is likely to be largely non-differential and may attenuate the observed associations, as individuals with higher childhood adiposity may be misclassified into lower body size categories, thereby reducing the contrast between exposure groups. Therefore, the true impact of early-life adiposity on later disease trajectories may be underestimated. In addition, the childhood-to-adulthood adiposity trajectories, constructed from a recalled childhood body size combined with a single adulthood BMI, inevitably simplify the complexity of weight change across the life course. Post-diagnosis data on medications and treatment adherence were limited in the UK Biobank, restricting our ability to account for treatment-related confounding and potential time-varying effects. Although baseline self-reported medication use is available, detailed information on treatment initiation, changes in therapy, dosage, and long-term adherence during follow-up is not comprehensively captured. Pharmacological interventions and clinical management may substantially influence disease progression and survival, and the absence of such longitudinal treatment data may therefore introduce residual confounding and affect the estimated transition risks between disease states. While baseline behaviors may not fully reflect long-term exposure, evidence suggests that substantial changes are uncommon without targeted interventions [45]. In addition, CKD cases were identified from hospital inpatient and death registry records, which may underestimate the true incidence of CKD, as many cases are diagnosed and managed in outpatient or primary care settings. Consequently, CKD cases captured in this study may represent relatively more severe disease, which may influence the estimated transition patterns involving CKD in the multi-state model. Finally, the cohort’s predominantly White European ancestry may restrict the generalizability of our findings to more diverse populations.

## Conclusion

This study demonstrated that preventing early and sustained adiposity and improving lifestyle behaviors are both essential for slowing progression along the multimorbidity pathway. T2D-related routes should be prioritized as key targets for risk reduction. Risk stratification can be strengthened by integrating body size trajectories, lifestyle, and CTI, which reflects inflammatory and insulin-resistance burden and highlights actionable biological pathways. Future work in more diverse populations with richer longitudinal and treatment data will refine causal interpretation and inform implementation.

## Supplementary Information

Additional file 1

## Data Availability

Data are available in a public, open access repository. The UK Biobank data are available on application to the UK Biobank (www.ukbiobank.ac.uk/) with access fees. However, data are available from the authors upon reasonable request and with the permission of the UK Biobank.
