# Supplementary material for: Life-course body size trajectories and the progression of cardio-renal-metabolic multimorbidity: a prospective UK biobank study
Source: Nutr Metab (Lond). 2026 Jun 4;23:92. doi: 10.1186/s12986-026-01148-7 (PMC13471649; doi:10.1186/s12986-026-01148-7)
Supplement: Supplementary file 1 [file 12986_2026_1148_MOESM1_ESM.docx]

**Supplementary material**

**Life-course Body Size Trajectories and the Progression of Cardio-Renal-Metabolic Multimorbidity: A Prospective UK Biobank Study**

**Table of Contents**

[**Table S1. Definition and scoring algorithm for seven Life’s Essential 8 (LE8) metrics included in this analysis.**	4](#_Toc225129704)

[**Table S2. Baseline characteristics of participants by childhood-to-adulthood body size trajectories.**	5](#_Toc225129705)

[**Table S3. Transition probabilities and expected length of stay (ELOS) for comorbidity-count progression trajectories at 10 and 15 years, stratified by childhood-to-adulthood body size trajectories.**	8](#_Toc225129706)

[**Table S4. Transition probabilities and expected length of stay (ELOS) for comorbidity-pattern progression trajectories at 10 and 15 years, stratified by childhood-to-adulthood body size trajectories.**	9](#_Toc225129707)

[**Table S5. Sex-stratified associations of childhood-to-adulthood body size trajectories with disease incidence and mortality outcomes.**	10](#_Toc225129708)

[**Table S6. Age-stratified associations of childhood-to-adulthood body size trajectories with disease incidence and mortality outcomes.**	13](#_Toc225129709)

[**Table S7. Associations of childhood-to-adulthood body size trajectories with disease incidence and mortality outcomes, excluding participants who experienced the corresponding outcome within the first 2 years of follow-up.**	16](#_Toc225129710)

[**Table S8. Associations of childhood-to-adulthood body size trajectories with disease incidence and mortality outcomes, excluding individuals missing covariates.**	17](#_Toc225129711)

[**Table S9. Associations of childhood-to-adulthood body size trajectories with disease incidence and mortality outcomes, excluding individuals with adult BMI< 18.5 kg/m².**	18](#_Toc225129712)

[**Table S10. Associations of childhood-to-adulthood body size trajectories with disease incidence and mortality outcomes** **after multiple imputation of missing covariates.**	19](#_Toc225129713)

[**Table S11. Associations of childhood-to-adulthood body size trajectories with disease incidence and mortality outcomes** **without adjustment for polygenic risk score for BMI (BMI-PRS).**	20](#_Toc225129714)

[**Figure S1. Flow diagram of the study population selection.**	21](#_Toc225129715)

[**Figure S2. Mediation effects of CTI on the associations between childhood-to-adulthood body size trajectories and the onset of different subtypes of CRMM.**	22](#_Toc225129716)

[**Figure S3. Age-stratified associations of childhood-to-adulthood body size trajectories with transitions along the comorbidity-count progression pathway (Healthy → First component disease → Double CRMM → Triple CRMM → Death) in multi-state models.**	23](#_Toc225129717)

[**Figure S4. Sex-stratified associations of childhood-to-adulthood body size trajectories with transitions along the comorbidity-count progression pathway (Healthy → First component disease → Double CRMM → Triple CRMM → Death) in multi-state models.**	24](#_Toc225129718)

[**Figure S5. Age-stratified associations of childhood-to-adulthood body size trajectories with transitions along the comorbidity-pattern progression pathway in multi-state models.**	25](#_Toc225129719)

[**Figure S6. Sex-stratified associations of childhood-to-adulthood body size trajectories with transitions along the comorbidity-pattern progression pathway in multi-state models.**	26](#_Toc225129720)

[**Figure S7. Associations of childhood-to-adulthood body size trajectories with transitions along the comorbidity-count progression pathway (Healthy → First component disease → Double CRMM → Triple CRMM → Death) in multi-state models, excluding individuals with adult BMI< 18.5 kg/m² (N = 392,556).**	27](#_Toc225129721)

[**Figure S8. Associations of childhood-to-adulthood body size trajectories with transitions along the comorbidity-pattern progression pathway in multi-state models, excluding individuals with adult BMI< 18.5 kg/m² (N = 392,556).**	28](#_Toc225129722)

[**Figure S9. Associations of childhood-to-adulthood body size trajectories with transitions along the comorbidity-count progression pathway (Healthy → First component disease → Double CRMM → Triple CRMM → Death) in multi-state models, excluding events occurring within the first two years of follow-up (N = 385,114).**	29](#_Toc225129723)

[**Figure S10. Associations of childhood-to-adulthood body size trajectories with transitions along the comorbidity-pattern progression pathway in multi-state models, excluding events occurring within the first two years of follow-up (N = 385,114).**	30](#_Toc225129724)

[**Figure S11. Associations of childhood-to-adulthood body size trajectories with transitions along the comorbidity-count progression pathway (Healthy → First component disease → Double CRMM → Triple CRMM → Death) in multi-state models, excluding individuals missing covariates (N = 382,638).**	31](#_Toc225129725)

[**Figure S12. Associations of childhood-to-adulthood body size trajectories with transitions along the comorbidity-pattern progression pathway in multi-state models, excluding individuals missing covariates (N = 382,638).**	32](#_Toc225129726)

[**Figure S13. Directed acyclic graph (DAG) depicting the assumed causal relationships among BMI polygenic risk score, childhood-to-adulthood body size trajectories, covariates, and cardio-renal-metabolic multimorbidity.**	33](#_Toc225129727)

**Table S1. Definition and scoring algorithm for seven Life’s Essential 8 (LE8) metrics included in this analysis.**

| **LE8 metrics** | **Method of measurement** | **Score Criteria** | **Points** |
| --- | --- | --- | --- |
| Diet | Self-reported daily intake of a DASH-style eating pattern | 95^th^ percentile (top/ideal diet) | 100 |
|  |  | 75^th^–94^th^ percentile | 80 |
|  |  | 50^th^–74^th^ percentile | 50 |
|  |  | 25^th^–49^th^ percentile | 25 |
|  |  | 1^st^–24^th^ percentile (bottom/least ideal quartile) | 0 |
| PA | Self-reported minutes of moderate or vigorous PA per week | ≥150 | 100 |
|  |  | 120–149 | 90 |
|  |  | 90–119 | 80 |
|  |  | 60–89 | 60 |
|  |  | 30–59 | 40 |
|  |  | 1–29 | 20 |
|  |  | 0 | 0 |
| Nicotine exposure | Self-reported use of cigarettes | Never smoker | 100 |
|  |  | Former smoker, quit ≥5 years | 75 |
|  |  | Former smoker, quit 1–<5 years | 50 |
|  |  | Former smoker, quit <1 year | 25 |
|  |  | Current smoker | 0 |
|  |  | Subtract 20 points (unless score is 0) for living with active indoor smoker in home | |
| Sleep health | Self-reported average hours of sleep per night | 7–<9 | 100 |
|  |  | 9–<10 | 90 |
|  |  | 6–<7 | 70 |
|  |  | 5–<6 or ≥10 | 40 |
|  |  | 4–<5 | 20 |
|  |  | <4 | 0 |

**Table S2. Baseline characteristics of participants by childhood-to-adulthood body size trajectories.**

| **Characteristics** | **Total** | **Thinner-to-normal** | **Thinner-to-overweight** | **Thinner-to-obesity** | **Average-to-normal** | **Average-to-overweight** | **Average-to- obesity** | **Plumper-to-normal** | **Plumper-to-overweight** | **Plumper-to-obesity** |
| --- | --- | --- | --- | --- | --- | --- | --- | --- | --- | --- |
| **Participants (n)** | 398,491 | 52,864 | 55,547 | 24,553 | 65,542 | 90,658 | 46,258 | 12,031 | 24,827 | 26,211 |
| **Age at recruitment (y)** | 56.61 (8.06) | 55.62 (8.25) | 57.21 (7.98) | 57.19 (7.88) | 56.17 (8.22) | 57.29 (8.00) | 57.04 (7.81) | 55.47 (7.96) | 56.25 (7.99) | 55.62 (7.84) |
| Missing | 0 (0.0) | 0 (0.0) | 0 (0.0) | 0 (0.0) | 0 (0.0) | 0 (0.0) | 0 (0.0) | 0 (0.0) | 0 (0.0) | 0 (0.0) |
| **Sex** |  |  |  |  |  |  |  |  |  |  |
| Female | 218,568 (54.8) | 31,843 (60.2) | 25,144 (45.3) | 12,865 (52.4) | 44,230 (67.5) | 42,357 (46.7) | 23,620 (51.1) | 9,035 (75.1) | 14,286 (57.5) | 15,188 (57.9) |
| Male | 179,923 (45.2) | 21,021 (39.8) | 30,403 (54.7) | 11,688 (47.6) | 21,312 (32.5) | 48,301 (53.3) | 22,638 (48.9) | 2,996 (24.9) | 10,541 (42.5) | 11,023 (42.1) |
| Missing | 0 (0.0) | 0 (0.0) | 0 (0.0) | 0 (0.0) | 0 (0.0) | 0 (0.0) | 0 (0.0) | 0 (0.0) | 0 (0.0) | 0 (0.0) |
| **Educational attainment** |  |  |  |  |  |  |  |  |  |  |
| Low | 113,379 (29.0) | 11,806 (22.7) | 18,017 (33.1) | 10,175 (42.4) | 14,711 (22.8) | 25,818 (29.0) | 15,291 (33.7) | 2,621 (22.1) | 6,935 (28.4) | 8,005 (31.1) |
| Moderate | 151,376 (38.7) | 19,522 (37.5) | 20,434 (37.5) | 89,26 (37.2) | 24,891 (38.6) | 35,065 (39.3) | 18,543 (40.9) | 4,336 (36.6) | 9,210 (37.7) | 10,449 (40.5) |
| High | 126,844 (32.4) | 20,688 (39.8) | 16,041 (29.4) | 48,80 (20.3) | 24,913 (38.6) | 28,258 (31.7) | 11,557 (25.5) | 4,878 (41.2) | 8,310 (34.0) | 7,319 (28.4) |
| Missing | 6,892 (0.0) | 848 (0.0) | 1,055 (0.0) | 572 (0.0) | 1,027 (0.0) | 1,517 (0.0) | 867 (0.0) | 196 (0.0) | 372 (0.0) | 438 (0.0) |
| **Ethnicity** |  |  |  |  |  |  |  |  |  |  |
| White | 377,638 (95.1) | 49,992 (94.9) | 52,032 (94.0) | 22,562 (92.2) | 62,655 (95.9) | 86,591 (95.8) | 43,790 (95.0) | 11,549 (96.3) | 23,636 (95.5) | 24,831 (95.1) |

**Table S2 (continued). Baseline characteristics of participants by childhood-to-adulthood body size trajectories.**

| **Characteristics** | **Total** | **Thinner-to-normal** | **Thinner-to-overweight** | **Thinner-to-obesity** | **Average-to-normal** | **Average-to-overweight** | **Average-to- obesity** | **Plumper-to-normal** | **Plumper-to-overweight** | **Plumper-to-obesity** |
| --- | --- | --- | --- | --- | --- | --- | --- | --- | --- | --- |
| Others | 19,614 (4.9) | 2,712 (5.1) | 3,327 (6.0) | 1,904 (7.8) | 2,711 (4.1) | 3,809 (4.2) | 2,317 (5.0) | 443 (3.7) | 1,101 (4.5) | 1,290 (4.9) |
| Missing | 1,239 (0.0) | 160 (0.0) | 188 (0.0) | 87 (0.0) | 176 (0.0) | 258 (0.0) | 151 (0.0) | 39 (0.0) | 90 (0.0) | 90 (0.0) |
| **TDI** |  |  |  |  |  |  |  |  |  |  |
| Low | 133,240 (33.5) | 18,523 (35.1) | 18,755 (33.8) | 6,513 (26.6) | 23,333 (35.6) | 32,137 (35.5) | 13,969 (30.2) | 4,151 (34.5) | 8,328 (33.6) | 7,531 (28.8) |
| Moderate | 132,195 (33.2) | 17,572 (33.3) | 18,475 (33.3) | 7,485 (30.5) | 21,643 (33.1) | 31,079 (34.3) | 15,329 (33.2) | 4,051 (33.7) | 8,239 (33.2) | 8,322 (31.8) |
| High | 132,568 (33.3) | 16,706 (31.6) | 18,249 (32.9) | 10,527 (42.9) | 20,493 (31.3) | 27,330 (30.2) | 16,894 (36.6) | 3,816 (31.8) | 8,236 (33.2) | 10,317 (39.4) |
| Missing | 488 (0.0) | 28 (0.0) | 73 (0.0) | 112 (0.0) | 66 (0.0) | 13 (0.0) | 24 (0.0) | 41 (0.0) | 28 (0.0) | 73 (0.0) |
| **Family history of CVD** |  |  |  |  |  |  |  |  |  |  |
| No | 165,317 (42.4) | 23,003 (44.3) | 22,497 (41.4) | 9,448 (39.7) | 28,861 (44.9) | 38,349 (43.2) | 18,482 (41.0) | 4,811 (40.8) | 9,851 (40.5) | 10,015 (39.2) |
| Yes | 224,535 (57.6) | 28,867 (55.7) | 31,799 (58.6) | 14,377 (60.3) | 35,465 (55.1) | 50,401 (56.8) | 26,621 (59.0) | 6,994 (59.2) | 14,457 (59.5) | 15,554 (60.8) |
| Missing | 8,639 (0.0) | 994 (0.0) | 1,251 (0.0) | 728 (0.0) | 1,216 (0.0) | 1,908 (0.0) | 1,155 (0.0) | 226 (0.0) | 519 (0.0) | 642 (0.0) |
| **Family history of diabetes** |  |  |  |  |  |  |  |  |  |  |
| No | 304,948 (78.2) | 42,165 (81.3) | 42,033 (77.4) | 17,087 (71.7) | 53,162 (82.6) | 70,717 (79.7) | 33,495 (74.3) | 9,403 (79.7) | 18,672 (76.8) | 18,214 (71.2) |
| Yes | 84,904 (21.8) | 9,705 (18.7) | 12,263 (22.6) | 6,738 (28.3) | 11,164 (17.4) | 18,033 (20.3) | 11,608 (25.7) | 2,402 (20.3) | 5,636 (23.2) | 7,355 (28.8) |

**Table S2 (continued). Baseline characteristics of participants by childhood-to-adulthood body size trajectories.**

| **Characteristics** | **Total** | **Thinner-to-normal** | **Thinner-to-overweight** | **Thinner-to-obesity** | **Average-to-normal** | **Average-to-overweight** | **Average-to- obesity** | **Plumper-to-normal** | **Plumper-to-overweight** | **Plumper-to-obesity** |
| --- | --- | --- | --- | --- | --- | --- | --- | --- | --- | --- |
| Missing | 8,639 (0.0) | 994 (0.0) | 1,251 (0.0) | 728 (0.0) | 1,216 (0.0) | 1,908 (0.0) | 1,155 (0.0) | 226 (0.0) | 519 (0.0) | 642 (0.0) |
| **Genetic risk score of BMI** |  |  |  |  |  |  |  |  |  |  |
| Low | 128,849 (33.3) | 25,782 (50.2) | 20170 (37.4) | 5,672 (24.0) | 26,485 (41.6) | 27,868 (31.6) | 9,459 (21.1) | 3,624 (31.2) | 5,898 (24.5) | 3,891 (15.4) |
| Moderate | 128,848 (33.3) | 15,965 (31.1) | 18,550 (34.4) | 7,911 (33.4) | 21,585 (33.9) | 30,861 (35.0) | 14,545 (32.5) | 3,952 (34.0) | 7,965 (33.0) | 7,514 (29.7) |
| High | 128,849 (33.3) | 9,610 (18.7) | 15,240 (28.2) | 10,082 (42.6) | 15,634 (24.5) | 29,370 (33.3) | 20,750 (46.4) | 4,045 (34.8) | 10,258 (42.5) | 13,860 (54.9) |
| Missing | 11,945 (0.0) | 1,507 (0.0) | 1,587 (0.0) | 888 (0.0) | 1,838 (0.0) | 2,559 (0.0) | 1,504 (0.0) | 410 (0.0) | 706 (0.0) | 946 (0.0) |
| **LE4 score groups** |  |  |  |  |  |  |  |  |  |  |
| Low | 199,229 (50.0) | 25,601 (48.4) | 27,747 (50.0) | 13,379 (54.5) | 31,152 (47.5) | 43,877 (48.4) | 24,226 (52.4) | 6,128 (50.9) | 12,677 (51.1) | 14,442 (55.1) |
| High | 199,262 (50.0) | 27,263 (51.6) | 27,800 (50.0) | 11,174 (45.5) | 34,390 (52.5) | 46,781 (51.6) | 22,032 (47.6) | 5,903 (49.1) | 12,150 (48.9) | 11,769 (44.9) |
| Missing | 0 (0.0) | 0 (0.0) | 0 (0.0) | 0 (0.0) | 0 (0.0) | 0 (0.0) | 0 (0.0) | 0 (0.0) | 0 (0.0) | 0 (0.0) |
| **CTI** | 1.41 (0.75) | 1.05 (0.68) | 1.59 (0.66) | 1.95 (0.63) | 0.93 (0.66) | 1.45 (0.67) | 1.88 (0.64) | 0.87 (0.66) | 1.38 (0.69) | 1.85 (0.66) |
| Missing | 78,447 (19.7) | 9,346 (17.7) | 10,302 (18.5) | 6,345 (25.9) | 11,514 (17.6) | 16,397 (18.1) | 10,854 (23.5) | 2,186 (18.2) | 4,587 (18.5) | 6,916 (26.4) |

Mean ± SD for continuous variables and percentage for categorical variables. LE4 indicates 4 lifestyle metrics from Life’s Essential 8.

Abbreviations: TDI, Townsend Deprivation Index; CVD, cardiovascular disease; BMI, body mass index; PA, physical activity; CTI, C-reactive protein-triglyceride glucose index.

**Table S3. Transition probabilities and expected length of stay (ELOS) for comorbidity-count progression trajectories at 10 and 15 years, stratified by childhood-to-adulthood body size trajectories.**

|  | **State occupation probabilities (%)** | | | | |  | **Expected length of stay (years)** | | | |
| --- | --- | --- | --- | --- | --- | --- | --- | --- | --- | --- |
|  | **Healthy** | **First component disease** | **Double CRMM** | **Triple CRMM** | **Death** |  | **Healthy** | **First component disease** | **Double CRMM** | **Triple CRMM** |
| **10 Years** |  |  |  |  |  |  |  |  |  |  |
| **Body size trajectories** |  |  |  |  |  |  |  |  |  |  |
| Thinner-to-normal | 88.91 | 7.04 | 0.46 | 0.01 | 3.58 |  | 9.20 | 0.51 | 0.03 | 0.00 |
| Thinner-to-overweight | 82.71 | 12.09 | 1.14 | 0.13 | 3.92 |  | 8.74 | 0.90 | 0.08 | 0.01 |
| Thinner-to-obesity | 73.34 | 18.62 | 2.84 | 0.28 | 4.92 |  | 8.03 | 1.42 | 0.19 | 0.02 |
| Average-to-normal | 89.52 | 6.27 | 0.38 | 0.02 | 3.81 |  | 9.25 | 0.46 | 0.02 | 0.00 |
| Average-to-overweight | 85.10 | 9.94 | 1.01 | 0.01 | 3.94 |  | 8.92 | 0.74 | 0.07 | 0.00 |
| Average-to- obesity | 77.26 | 15.92 | 2.15 | 0.26 | 4.41 |  | 8.33 | 1.20 | 0.14 | 0.02 |
| Plumper-to-normal | 90.36 | 5.70 | 0.22 | 0.00 | 3.72 |  | 9.31 | 0.41 | 0.02 | 0.00 |
| Plumper-to-overweight | 85.75 | 9.42 | 0.84 | 0.12 | 3.87 |  | 8.96 | 0.70 | 0.06 | 0.01 |
| Plumper-to-obesity | 77.74 | 15.51 | 2.02 | 0.32 | 4.41 |  | 8.36 | 1.18 | 0.14 | 0.02 |
| **15 Years** |  |  |  |  |  |  |  |  |  |  |
| **Body size trajectories** |  |  |  |  |  |  |  |  |  |  |
| Thinner-to-normal | 81.56 | 9.88 | 1.27 | 0.01 | 7.28 |  | 12.98 | 1.11 | 0.13 | 0.00 |
| Thinner-to-overweight | 72.66 | 16.71 | 2.19 | 0.77 | 7.67 |  | 12.00 | 1.91 | 0.23 | 0.07 |
| Thinner-to-obesity | 60.91 | 24.49 | 4.55 | 0.71 | 9.35 |  | 10.59 | 2.85 | 0.49 | 0.07 |
| Average-to-normal | 82.26 | 9.26 | 1.26 | 0.11 | 7.11 |  | 13.08 | 1.04 | 0.12 | 0.01 |
| Average-to-overweight | 75.55 | 13.88 | 2.08 | 0.09 | 8.40 |  | 12.34 | 1.59 | 0.21 | 0.01 |
| Average-to- obesity | 65.81 | 20.28 | 4.09 | 0.37 | 9.45 |  | 11.18 | 2.38 | 0.42 | 0.04 |
| Plumper-to-normal | 83.31 | 9.16 | 0.42 | 0.00 | 7.10 |  | 13.17 | 0.99 | 0.04 | 0.00 |
| Plumper-to-overweight | 76.83 | 13.59 | 2.03 | 0.12 | 7.43 |  | 12.45 | 1.53 | 0.21 | 0.01 |
| Plumper-to-obesity | 66.43 | 20.25 | 4.83 | 0.52 | 7.97 |  | 11.22 | 2.37 | 0.47 | 0.05 |

Abbreviations: CRMM, cardio-renal-metabolic multimorbidity.

**Table S4. Transition probabilities and expected length of stay (ELOS) for comorbidity-pattern progression trajectories at 10 and 15 years, stratified by childhood-to-adulthood body size trajectories.**

|  | **State occupation probabilities (%)** | | | | | | | |  | **Expected length of stay (years)** | | | | | | |
| --- | --- | --- | --- | --- | --- | --- | --- | --- | --- | --- | --- | --- | --- | --- | --- | --- |
|  | **Healthy** | **CVD** | **T2D** | **CKD** | **CVD- T2D** | **CVD- CKD** | **T2D-CKD** | **CRMM** |  | **Healthy** | **CVD** | **T2D** | **CKD** | **CVD- T2D** | **CVD- CKD** | **T2D-CKD** |
| **10 Years** |  |  |  |  |  |  |  |  |  |  |  |  |  |  |  |  |
| **Body size trajectories** |  |  |  |  |  |  |  |  |  |  |  |  |  |  |  |  |
| Thinner-to-normal | 91.76 | 5.28 | 1.41 | 0.97 | 0.22 | 0.26 | 0.09 | 0.01 |  | 9.40 | 0.39 | 0.11 | 0.06 | 0.01 | 0.02 | 0.01 |
| Thinner-to-overweight | 85.43 | 7.48 | 4.24 | 1.31 | 0.64 | 0.53 | 0.24 | 0.14 |  | 8.94 | 0.56 | 0.31 | 0.09 | 0.04 | 0.03 | 0.02 |
| Thinner-to-obesity | 76.15 | 7.56 | 10.77 | 1.88 | 1.70 | 0.75 | 0.85 | 0.34 |  | 8.24 | 0.58 | 0.81 | 0.13 | 0.11 | 0.05 | 0.06 |
| Average-to-normal | 92.60 | 4.83 | 1.13 | 0.96 | 0.13 | 0.27 | 0.07 | 0.02 |  | 9.47 | 0.35 | 0.08 | 0.06 | 0.01 | 0.02 | 0.00 |
| Average-to-overweight | 87.97 | 6.68 | 2.73 | 1.41 | 0.52 | 0.50 | 0.16 | 0.03 |  | 9.12 | 0.50 | 0.20 | 0.09 | 0.03 | 0.03 | 0.01 |
| Average-to- obesity | 79.94 | 7.23 | 8.17 | 1.90 | 1.21 | 0.64 | 0.62 | 0.30 |  | 8.53 | 0.55 | 0.62 | 0.13 | 0.08 | 0.04 | 0.04 |
| Plumper-to-normal | 93.35 | 4.37 | 1.09 | 0.86 | 0.15 | 0.15 | 0.02 | 0.00 |  | 9.52 | 0.31 | 0.08 | 0.06 | 0.01 | 0.01 | 0.00 |
| Plumper-to-overweight | 88.57 | 6.19 | 2.67 | 1.44 | 0.47 | 0.38 | 0.15 | 0.13 |  | 9.17 | 0.46 | 0.20 | 0.10 | 0.03 | 0.03 | 0.01 |
| Plumper-to-obesity | 80.40 | 6.65 | 8.58 | 1.66 | 1.29 | 0.44 | 0.66 | 0.32 |  | 8.55 | 0.50 | 0.65 | 0.11 | 0.09 | 0.03 | 0.04 |
| **15 Years** |  |  |  |  |  |  |  |  |  |  |  |  |  |  |  |  |
| **Body size trajectories** |  |  |  |  |  |  |  |  |  |  |  |  |  |  |  |  |
| Thinner-to-normal | 86.84 | 7.90 | 1.71 | 2.14 | 0.61 | 0.58 | 0.20 | 0.01 |  | 13.54 | 0.88 | 0.20 | 0.22 | 0.06 | 0.06 | 0.02 |
| Thinner-to-overweight | 77.63 | 10.44 | 5.69 | 2.93 | 1.32 | 1.00 | 0.35 | 0.63 |  | 12.52 | 1.20 | 0.65 | 0.29 | 0.13 | 0.10 | 0.04 |
| Thinner-to-obesity | 65.77 | 10.37 | 13.88 | 3.79 | 2.36 | 1.17 | 1.86 | 0.79 |  | 11.12 | 1.20 | 1.62 | 0.39 | 0.26 | 0.13 | 0.19 |
| Average-to-normal | 87.85 | 7.38 | 1.19 | 2.16 | 0.15 | 0.67 | 0.54 | 0.06 |  | 13.67 | 0.82 | 0.15 | 0.21 | 0.02 | 0.07 | 0.04 |
| Average-to-overweight | 80.68 | 10.08 | 3.58 | 3.08 | 0.95 | 1.06 | 0.48 | 0.09 |  | 12.88 | 1.13 | 0.42 | 0.31 | 0.10 | 0.11 | 0.05 |
| Average-to- obesity | 70.49 | 9.96 | 10.35 | 3.57 | 2.63 | 1.19 | 1.45 | 0.37 |  | 11.68 | 1.15 | 1.22 | 0.37 | 0.27 | 0.12 | 0.15 |
| Plumper-to-normal | 89.21 | 6.84 | 1.57 | 1.89 | 0.15 | 0.15 | 0.20 | 0.00 |  | 13.61 | 0.73 | 0.17 | 0.19 | 0.02 | 0.02 | 0.02 |
| Plumper-to-overweight | 81.90 | 9.01 | 3.45 | 3.11 | 1.01 | 1.00 | 0.38 | 0.13 |  | 12.95 | 1.01 | 0.40 | 0.31 | 0.10 | 0.10 | 0.04 |
| Plumper-to-obesity | 71.19 | 8.48 | 11.05 | 3.46 | 2.28 | 1.84 | 1.16 | 0.54 |  | 11.73 | 1.00 | 1.30 | 0.35 | 0.24 | 0.16 | 0.12 |

Abbreviations: CRMM, cardio-renal-metabolic multimorbidity; CVD, cardiovascular disease; T2D, type 2 diabetes; CKD, chronic kidney disease.

**Table S5. Sex-stratified associations of childhood-to-adulthood body size trajectories with disease incidence and mortality outcomes.**

|  | **First component disease**  **HR (95% CI)** | **Double CRMM**  **HR (95% CI)** | **CVD-T2D**  **HR (95% CI)** | **CVD-CKD**  **HR (95% CI)** | **T2D-CKD**  **HR (95% CI)** | **Triple CRMM**  **HR (95% CI)** | **All-cause Mortality**  **HR (95% CI)** |
| --- | --- | --- | --- | --- | --- | --- | --- |
| **Male** |  |  |  |  |  |  |  |
| **Body size trajectories** |  |  |  |  |  |  |  |
| Thinner-to-normal | 1.06 (1.01, 1.11) | 1.14 (1.00, 1.29) | 1.24 (1.03, 1.50) | 1.07 (0.89, 1.28) | 1.25 (0.93, 1.67) | 1.30 (0.84, 2.03) | 0.94 (0.89, 1.00) |
| Thinner-to-overweight | 1.49 (1.43, 1.55) | 1.97 (1.77, 2.19) | 2.63 (2.25, 3.07) | 1.42 (1.22, 1.65) | 2.23 (1.75, 2.84) | 2.34 (1.62, 3.39) | 0.86 (0.81, 0.91) |
| Thinner-to-obesity | 2.44 (2.34, 2.55) | 4.52 (4.05, 5.03) | 6.55 (5.61, 7.65) | 2.63 (2.23, 3.09) | 7.32 (5.78, 9.27) | 7.80 (5.44, 11.17) | 1.14 (1.07, 1.21) |
| Average-to-normal | Ref | Ref | Ref | Ref | Ref | Ref | Ref |
| Average-to-overweight | 1.34 (1.29, 1.39) | 1.73 (1.56, 1.92) | 2.09 (1.80, 2.44) | 1.48 (1.29, 1.71) | 1.95 (1.54, 2.47) | 2.23 (1.56, 3.19) | 0.87 (0.82, 0.91) |
| Average-to-obesity | 2.19 (2.10, 2.28) | 3.87 (3.50, 4.29) | 5.71 (4.91, 6.63) | 2.41 (2.07, 2.80) | 6.08 (4.84, 7.64) | 7.07 (4.99, 10.01) | 1.03 (0.98, 1.09) |
| Plumper-to-normal | 0.95 (0.86, 1.04) | 0.90 (0.67, 1.19) | 1.06 (0.72, 1.58) | 0.79 (0.51, 1.21) | 1.23 (0.69, 2.21) | 1.34 (0.57, 3.19) | 1.04 (0.92, 1.17) |
| Plumper-to-overweight | 1.35 (1.28, 1.43) | 1.72 (1.50, 1.98) | 2.05 (1.69, 2.50) | 1.48 (1.21, 1.80) | 2.29 (1.70, 3.07) | 2.48 (1.59, 3.87) | 0.94 (0.87, 1.01) |
| Plumper-to-obesity | 2.35 (2.24, 2.46) | 4.25 (3.80, 4.76) | 6.48 (5.52, 7.59) | 2.37 (1.98, 2.82) | 6.43 (5.03, 8.22) | 7.37 (5.08, 10.68) | 1.15 (1.07, 1.23) |

**Table S5 (continued). Sex-stratified associations of childhood-to-adulthood body size trajectories with disease incidence and mortality outcomes.**

|  | **First component disease**  **HR (95% CI)** | **Double CRMM**  **HR (95% CI)** | **CVD-T2D**  **HR (95% CI)** | **CVD-CKD**  **HR (95% CI)** | **T2D-CKD**  **HR (95% CI)** | **Triple CRMM**  **HR (95% CI)** | **All-cause Mortality**  **HR (95% CI)** |
| --- | --- | --- | --- | --- | --- | --- | --- |
| **Female** |  |  |  |  |  |  |  |
| **Body size trajectories** |  |  |  |  |  |  |  |
| Thinner-to-normal | 1.12 (1.07, 1.17) | 1.20 (1.04, 1.39) | 1.45 (1.14, 1.85) | 1.02 (0.84, 1.24) | 1.48 (1.09, 2.02) | 1.32 (0.79, 2.21) | 1.02 (0.96, 1.09) |
| Thinner-to-overweight | 1.73 (1.66, 1.81) | 2.62 (2.32, 2.96) | 4.25 (3.49, 5.19) | 1.80 (1.53, 2.13) | 3.72 (2.88, 4.81) | 4.20 (2.79, 6.33) | 0.98 (0.92, 1.04) |
| Thinner-to-obesity | 2.96 (2.83, 3.10) | 5.50 (4.87, 6.20) | 8.90 (7.31, 10.82) | 3.07 (2.59, 3.64) | 10.83 (8.48, 13.82) | 10.41 (6.99, 15.51) | 1.25 (1.16, 1.34) |
| Average-to-normal | Ref | Ref | Ref | Ref | Ref | Ref | Ref |
| Average-to-overweight | 1.40 (1.34, 1.46) | 1.78 (1.58, 2.01) | 2.44 (1.99, 2.98) | 1.44 (1.23, 1.68) | 2.21 (1.71, 2.87) | 2.39 (1.57, 3.61) | 0.97 (0.92, 1.02) |
| Average-to-obesity | 2.50 (2.39, 2.60) | 4.30 (3.83, 4.83) | 6.75 (5.57, 8.17) | 2.69 (2.30, 3.15) | 7.88 (6.19, 10.01) | 7.87 (5.32, 11.64) | 1.15 (1.08, 1.22) |
| Plumper-to-normal | 0.99 (0.92, 1.07) | 0.85 (0.66, 1.10) | 0.86 (0.55, 1.33) | 0.77 (0.55, 1.09) | 0.88 (0.50, 1.55) | 0.50 (0.15, 1.63) | 1.20 (1.10, 1.32) |
| Plumper-to-overweight | 1.49 (1.41, 1.57) | 2.10 (1.81, 2.44) | 2.77 (2.18, 3.53) | 1.62 (1.32, 2.00) | 2.77 (2.03, 3.77) | 2.38 (1.41, 4.01) | 1.10 (1.02, 1.18) |
| Plumper-to-obesity | 2.69 (2.57, 2.82) | 4.47 (3.94, 5.06) | 6.63 (5.41, 8.12) | 2.74 (2.29, 3.28) | 9.15 (7.12, 11.75) | 8.41 (5.57, 12.69) | 1.31 (1.22, 1.40) |

Models were adjusted for age, educational attainment, ethnicity and Townsend Deprivation Index, family history of CVD, family history of T2D, polygenic risk score for BMI and LE4 score. LE4 indicates 4 lifestyle metrics from Life’s Essential 8. First component disease denotes the first occurrence of any one of CVD, T2D, or CKD during follow-up. Double CRMM denotes the first attainment of any two of these conditions (i.e., T2D-CVD, CVD-CKD, or T2D-CKD); the event date is the date of the second qualifying diagnosis. Triple CRMM denotes the occurrence of all three conditions; the event date is the date of the third qualifying diagnosis.

Abbreviations: CRMM, cardio-renal-metabolic multimorbidity; CVD, cardiovascular disease; T2D, type 2 diabetes; CKD, chronic kidney disease; HR, hazard ratio; CI, confidence interval; Ref, reference.

**Table S6. Age-stratified associations of childhood-to-adulthood body size trajectories with disease incidence and mortality outcomes.**

|  | **First component disease**  **HR (95% CI)** | **Double CRMM**  **HR (95% CI)** | **CVD-T2D**  **HR (95% CI)** | **CVD-CKD**  **HR (95% CI)** | **T2D-CKD**  **HR (95% CI)** | **Triple CRMM**  **HR (95% CI)** | **All-cause Mortality**  **HR (95% CI)** |
| --- | --- | --- | --- | --- | --- | --- | --- |
| **<60 years** |  |  |  |  |  |  |  |
| **Body size trajectories** |  |  |  |  |  |  |  |
| Thinner-to-normal | 1.14 (1.07, 1.20) | 1.18 (0.98, 1.41) | 1.28 (0.99, 1.65) | 1.15 (0.87, 1.51) | 1.35 (0.90, 2.02) | 1.63 (0.84, 3.16) | 1.04 (0.97, 1.13) |
| Thinner-to-overweight | 1.73 (1.64, 1.82) | 2.37 (2.03, 2.77) | 3.10 (2.50, 3.83) | 1.57 (1.23, 2.01) | 2.84 (2.01, 4.01) | 2.95 (1.65, 5.27) | 0.90 (0.83, 0.97) |
| Thinner-to-obesity | 3.21 (3.05, 3.38) | 5.67 (4.87, 6.59) | 7.88 (6.40, 9.72) | 3.00 (2.33, 3.86) | 9.41 (6.79, 13.05) | 10.14 (5.84, 17.61) | 1.24 (1.14, 1.36) |
| Average-to-normal | Ref | Ref | Ref | Ref | Ref | Ref | Ref |
| Average-to-overweight | 1.46 (1.40, 1.53) | 1.80 (1.55, 2.10) | 2.33 (1.88, 2.87) | 1.33 (1.05, 1.69) | 1.77 (1.25, 2.51) | 2.11 (1.18, 3.75) | 0.91 (0.85, 0.98) |
| Average-to-obesity | 2.77 (2.64, 2.91) | 4.43 (3.83, 5.13) | 6.34 (5.17, 7.77) | 2.21 (1.74, 2.81) | 6.16 (4.45, 8.51) | 6.49 (3.75, 11.22) | 1.10 (1.02, 1.19) |
| Plumper-to-normal | 0.96 (0.87, 1.06) | 0.88 (0.63, 1.24) | 0.90 (0.55, 1.46) | 0.74 (0.43, 1.27) | 0.57 (0.22, 1.43) | NE | 1.12 (1.00, 1.27) |
| Plumper-to-overweight | 1.52 (1.43, 1.61) | 1.93 (1.60, 2.34) | 2.27 (1.75, 2.95) | 1.32 (0.96, 1.81) | 2.60 (1.73, 3.89) | 1.99 (0.96, 4.12) | 0.95 (0.86, 1.05) |
| Plumper-to-obesity | 2.99 (2.84, 3.15) | 5.00 (4.29, 5.83) | 7.15 (5.79, 8.82) | 2.35 (1.81, 3.05) | 7.79 (5.59, 10.84) | 7.92 (4.52, 13.88) | 1.25 (1.15, 1.36) |

**Table S6 (continued). Age-stratified associations of childhood-to-adulthood body size trajectories with disease incidence and mortality outcomes.**

|  | **First component disease**  **HR (95% CI)** | **Double CRMM**  **HR (95% CI)** | **CVD-T2D**  **HR (95% CI)** | **CVD-CKD**  **HR (95% CI)** | **T2D-CKD**  **HR (95% CI)** | **Triple CRMM**  **HR (95% CI)** | **All-cause Mortality**  **HR (95% CI)** |
| --- | --- | --- | --- | --- | --- | --- | --- |
| **≥60 years** |  |  |  |  |  |  |  |
| **Body size trajectories** |  |  |  |  |  |  |  |
| Thinner-to-normal | 1.08 (1.04, 1.13) | 1.18 (1.06, 1.32) | 1.37 (1.14, 1.64) | 1.03 (0.89, 1.20) | 1.38 (1.08, 1.77) | 1.23 (0.83, 1.82) | 0.95 (0.91, 1.00) |
| Thinner-to-overweight | 1.55 (1.49, 1.61) | 2.17 (1.97, 2.38) | 3.16 (2.73, 3.67) | 1.58 (1.40, 1.80) | 2.85 (2.32, 3.49) | 3.00 (2.20, 4.09) | 0.92 (0.87, 0.96) |
| Thinner-to-obesity | 2.42 (2.33, 2.52) | 4.69 (4.27, 5.16) | 7.24 (6.23, 8.41) | 2.80 (2.45, 3.20) | 8.73 (7.16, 10.65) | 8.53 (6.28, 11.57) | 1.17 (1.11, 1.24) |
| Average-to-normal | Ref | Ref | Ref | Ref | Ref | Ref | Ref |
| Average-to-overweight | 1.34 (1.30, 1.39) | 1.78 (1.62, 1.94) | 2.23 (1.92, 2.58) | 1.53 (1.36, 1.73) | 2.25 (1.84, 2.74) | 2.41 (1.78, 3.27) | 0.91 (0.88, 0.95) |
| Average-to-obesity | 2.13 (2.05, 2.20) | 3.99 (3.65, 4.37) | 6.13 (5.30, 7.08) | 2.66 (2.35, 3.00) | 7.28 (6.00, 8.83) | 7.85 (5.85, 10.55) | 1.09 (1.04, 1.14) |
| Plumper-to-normal | 0.99 (0.91, 1.07) | 0.86 (0.68, 1.09) | 0.98 (0.67, 1.41) | 0.78 (0.57, 1.07) | 1.19 (0.76, 1.87) | 1.17 (0.58, 2.38) | 1.14 (1.04, 1.25) |
| Plumper-to-overweight | 1.39 (1.33, 1.46) | 1.90 (1.68, 2.14) | 2.38 (1.97, 2.87) | 1.63 (1.39, 1.91) | 2.51 (1.95, 3.23) | 2.64 (1.81, 3.86) | 1.04 (0.98, 1.11) |
| Plumper-to-obesity | 2.24 (2.15, 2.34) | 4.11 (3.71, 4.55) | 6.28 (5.37, 7.36) | 2.62 (2.27, 3.03) | 7.65 (6.21, 9.41) | 7.86 (5.73, 10.79) | 1.21 (1.14, 1.29) |

Models were adjusted for sex, educational attainment, ethnicity and Townsend Deprivation Index, family history of CVD, family history of T2D, polygenic risk score for BMI and LE4 score. LE4 indicates 4 lifestyle metrics from Life’s Essential 8. NE, not estimable due to sparse events or (quasi-)complete separation in the Cox model. First component disease denotes the first occurrence of any one of CVD, T2D, or CKD during follow-up. Double CRMM denotes the first attainment of any two of these conditions (i.e., T2D-CVD, CVD-CKD, or T2D-CKD); the event date is the date of the second qualifying diagnosis. Triple CRMM denotes the occurrence of all three conditions; the event date is the date of the third qualifying diagnosis.

Abbreviations: CRMM, cardio-renal-metabolic multimorbidity; CVD, cardiovascular disease; T2D, type 2 diabetes; CKD, chronic kidney disease; HR, hazard ratio; CI, confidence interval; Ref, reference.

**Table S7. Associations of childhood-to-adulthood body size trajectories with disease incidence and mortality outcomes, excluding participants who experienced the corresponding outcome within the first 2 years of follow-up.**

|  | **First component disease**  **HR (95% CI)** | **Double CRMM**  **HR (95% CI)** | **CVD-T2D**  **HR (95% CI)** | **CVD-CKD**  **HR (95% CI)** | **T2D-CKD**  **HR (95% CI)** | **Triple CRMM**  **HR (95% CI)** | **All-cause Mortality**  **HR (95% CI)** |
| --- | --- | --- | --- | --- | --- | --- | --- |
| **Body size trajectories** |  |  |  |  |  |  |  |
| Thinner-to-normal | 1.09 (1.05, 1.13) | 1.19 (1.08, 1.31) | 1.38 (1.18, 1.61) | 1.06 (0.93, 1.21) | 1.37 (1.11, 1.70) | 1.34 (0.96, 1.88) | 0.98 (0.94, 1.03) |
| Thinner-to-overweight | 1.59 (1.54, 1.64) | 2.23 (2.05, 2.41) | 3.24 (2.85, 3.68) | 1.58 (1.41, 1.77) | 2.85 (2.39, 3.41) | 3.02 (2.29, 3.98) | 0.92 (0.88, 0.96) |
| Thinner-to-obesity | 2.67 (2.58, 2.77) | 4.94 (4.55, 5.36) | 7.63 (6.72, 8.66) | 2.86 (2.55, 3.22) | 8.94 (7.53, 10.60) | 9.05 (6.92, 11.84) | 1.21 (1.15, 1.27) |
| Average-to-normal | Ref | Ref | Ref | Ref | Ref | Ref | Ref |
| Average-to-overweight | 1.38 (1.34, 1.43) | 1.81 (1.67, 1.95) | 2.35 (2.07, 2.66) | 1.50 (1.35, 1.66) | 2.16 (1.81, 2.57) | 2.39 (1.82, 3.13) | 0.93 (0.89, 0.96) |
| Average-to- obesity | 2.31 (2.24, 2.38) | 4.09 (3.78, 4.42) | 6.33 (5.60, 7.16) | 2.56 (2.30, 2.85) | 7.01 (5.93, 8.28) | 7.59 (5.85, 9.86) | 1.10 (1.05, 1.14) |
| Plumper-to-normal | 0.98 (0.92, 1.04) | 0.87 (0.71, 1.05) | 0.96 (0.71, 1.30) | 0.78 (0.59, 1.02) | 1.00 (0.66, 1.50) | 0.85 (0.42, 1.71) | 1.13 (1.05, 1.22) |
| Plumper-to-overweight | 1.42 (1.37, 1.48) | 1.91 (1.73, 2.12) | 2.41 (2.06, 2.82) | 1.56 (1.36, 1.81) | 2.54 (2.05, 3.14) | 2.51 (1.79, 3.52) | 1.02 (0.96, 1.07) |
| Plumper-to-obesity | 2.50 (2.41, 2.59) | 4.39 (4.03, 4.78) | 6.88 (6.04, 7.83) | 2.55 (2.25, 2.89) | 7.73 (6.48, 9.22) | 7.98 (6.05, 10.53) | 1.24 (1.18, 1.30) |

Models were adjusted for age, sex, educational attainment, ethnicity and Townsend Deprivation Index, family history of CVD, family history of T2D, polygenic risk score for BMI and LE4 score. LE4 indicates 4 lifestyle metrics from Life’s Essential 8. First component disease denotes the first occurrence of any one of CVD, T2D, or CKD during follow-up. Double CRMM denotes the first attainment of any two of these conditions (i.e., T2D-CVD, CVD-CKD, or T2D-CKD); the event date is the date of the second qualifying diagnosis. Triple CRMM denotes the occurrence of all three conditions; the event date is the date of the third qualifying diagnosis.

Abbreviations: CRMM, cardio-renal-metabolic multimorbidity; CVD, cardiovascular disease; T2D, type 2 diabetes; CKD, chronic kidney disease; HR, hazard ratio; CI, confidence interval; Ref, reference.

**Table S8. Associations of childhood-to-adulthood body size trajectories with disease incidence and mortality outcomes, excluding individuals missing covariates.**

|  | **First component disease**  **HR (95% CI)** | **Double CRMM**  **HR (95% CI)** | **CVD-T2D**  **HR (95% CI)** | **CVD-CKD**  **HR (95% CI)** | **T2D-CKD**  **HR (95% CI)** | **Triple CRMM**  **HR (95% CI)** | **All-cause Mortality**  **HR (95% CI)** |
| --- | --- | --- | --- | --- | --- | --- | --- |
| **Body size trajectories** |  |  |  |  |  |  |  |
| Thinner-to-normal | 1.09 (1.05, 1.12) | 1.15 (1.05, 1.27) | 1.32 (1.14, 1.54) | 1.03 (0.90, 1.18) | 1.34 (1.08, 1.66) | 1.32 (0.94, 1.87) | 0.97 (0.93, 1.01) |
| Thinner-to-overweight | 1.60 (1.56, 1.65) | 2.19 (2.02, 2.37) | 3.13 (2.76, 3.54) | 1.56 (1.39, 1.74) | 2.85 (2.38, 3.41) | 3.06 (2.31, 4.05) | 0.90 (0.87, 0.94) |
| Thinner-to-obesity | 2.71 (2.62, 2.79) | 4.96 (4.57, 5.38) | 7.56 (6.68, 8.55) | 2.83 (2.51, 3.19) | 8.88 (7.47, 10.55) | 9.08 (6.91, 11.94) | 1.18 (1.13, 1.24) |
| Average-to-normal | Ref | Ref | Ref | Ref | Ref | Ref | Ref |
| Average-to-overweight | 1.38 (1.34, 1.42) | 1.76 (1.63, 1.91) | 2.23 (1.97, 2.52) | 1.48 (1.33, 1.65) | 2.14 (1.79, 2.55) | 2.39 (1.81, 3.14) | 0.91 (0.88, 0.95) |
| Average-to- obesity | 2.34 (2.28, 2.41) | 4.05 (3.74, 4.37) | 6.13 (5.44, 6.92) | 2.54 (2.28, 2.84) | 6.97 (5.89, 8.24) | 7.67 (5.88, 10.01) | 1.08 (1.04, 1.13) |
| Plumper-to-normal | 0.97 (0.92, 1.04) | 0.86 (0.71, 1.04) | 0.95 (0.70, 1.28) | 0.77 (0.59, 1.02) | 0.99 (0.66, 1.50) | 0.90 (0.45, 1.80) | 1.14 (1.06, 1.22) |
| Plumper-to-overweight | 1.44 (1.39, 1.50) | 1.90 (1.71, 2.10) | 2.34 (2.01, 2.73) | 1.56 (1.35, 1.80) | 2.58 (2.08, 3.21) | 2.56 (1.82, 3.60) | 1.01 (0.96, 1.07) |
| Plumper-to-obesity | 2.55 (2.46, 2.63) | 4.32 (3.97, 4.71) | 6.59 (5.80, 7.48) | 2.54 (2.23, 2.88) | 7.62 (6.38, 9.12) | 7.97 (6.01, 10.56) | 1.21 (1.15, 1.28) |

Models were adjusted for age, sex, educational attainment, ethnicity and Townsend Deprivation Index, family history of CVD, family history of T2D, polygenic risk score for BMI and LE4 score. LE4 indicates 4 lifestyle metrics from Life’s Essential 8. First component disease denotes the first occurrence of any one of CVD, T2D, or CKD during follow-up. Double CRMM denotes the first attainment of any two of these conditions (i.e., T2D-CVD, CVD-CKD, or T2D-CKD); the event date is the date of the second qualifying diagnosis. Triple CRMM denotes the occurrence of all three conditions; the event date is the date of the third qualifying diagnosis.

Abbreviations: CRMM, cardio-renal-metabolic multimorbidity; CVD, cardiovascular disease; T2D, type 2 diabetes; CKD, chronic kidney disease; HR, hazard ratio; CI, confidence interval; Ref, reference.

**Table S9. Associations of childhood-to-adulthood body size trajectories with disease incidence and mortality outcomes, excluding individuals with adult BMI< 18.5 kg/m².**

|  | **First component disease**  **HR (95% CI)** | **Double CRMM**  **HR (95% CI)** | **CVD-T2D**  **HR (95% CI)** | **CVD-CKD**  **HR (95% CI)** | **T2D-CKD**  **HR (95% CI)** | **Triple CRMM**  **HR (95% CI)** | **All-cause Mortality**  **HR (95% CI)** |
| --- | --- | --- | --- | --- | --- | --- | --- |
| **Body size trajectories** |  |  |  |  |  |  |  |
| Thinner-to-normal | 1.09 (1.06, 1.13) | 1.17 (1.07, 1.29) | 1.33 (1.14, 1.54) | 1.06 (0.93, 1.21) | 1.35 (1.09, 1.67) | 1.29 (0.92, 1.81) | 0.98 (0.94, 1.02) |
| Thinner-to-overweight | 1.61 (1.57, 1.66) | 2.22 (2.05, 2.41) | 3.17 (2.81, 3.59) | 1.58 (1.41, 1.76) | 2.81 (2.36, 3.35) | 2.95 (2.24, 3.89) | 0.93 (0.89, 0.96) |
| Thinner-to-obesity | 2.71 (2.62, 2.79) | 4.97 (4.59, 5.39) | 7.53 (6.66, 8.51) | 2.84 (2.53, 3.20) | 8.81 (7.43, 10.44) | 8.81 (6.75, 11.51) | 1.21 (1.16, 1.27) |
| Average-to-normal | Ref | Ref | Ref | Ref | Ref | Ref | Ref |
| Average-to-overweight | 1.39 (1.35, 1.43) | 1.79 (1.65, 1.93) | 2.28 (2.02, 2.58) | 1.49 (1.34, 1.66) | 2.11 (1.78, 2.51) | 2.32 (1.78, 3.04) | 0.93 (0.90, 0.96) |
| Average-to- obesity | 2.35 (2.28, 2.42) | 4.11 (3.80, 4.43) | 6.24 (5.54, 7.03) | 2.55 (2.29, 2.84) | 6.89 (5.84, 8.13) | 7.43 (5.73, 9.63) | 1.11 (1.06, 1.15) |
| Plumper-to-normal | 0.96 (0.90, 1.02) | 0.86 (0.71, 1.04) | 0.96 (0.71, 1.29) | 0.76 (0.58, 1.00) | 0.99 (0.66, 1.48) | 0.84 (0.42, 1.69) | 1.13 (1.06, 1.22) |
| Plumper-to-overweight | 1.43 (1.38, 1.49) | 1.90 (1.72, 2.10) | 2.36 (2.02, 2.75) | 1.55 (1.35, 1.79) | 2.51 (2.03, 3.10) | 2.45 (1.75, 3.43) | 1.03 (0.98, 1.08) |
| Plumper-to-obesity | 2.54 (2.45, 2.62) | 4.40 (4.04, 4.79) | 6.71 (5.92, 7.62) | 2.56 (2.25, 2.90) | 7.63 (6.40, 9.09) | 7.81 (5.93, 10.28) | 1.24 (1.18, 1.30) |

Models were adjusted for age, sex, educational attainment, ethnicity and Townsend Deprivation Index, family history of CVD, family history of T2D, polygenic risk score for BMI and LE4 score. LE4 indicates 4 lifestyle metrics from Life’s Essential 8. First component disease denotes the first occurrence of any one of CVD, T2D, or CKD during follow-up. Double CRMM denotes the first attainment of any two of these conditions (i.e., T2D-CVD, CVD-CKD, or T2D-CKD); the event date is the date of the second qualifying diagnosis. Triple CRMM denotes the occurrence of all three conditions; the event date is the date of the third qualifying diagnosis.

Abbreviations: CRMM, cardio-renal-metabolic multimorbidity; CVD, cardiovascular disease; T2D, type 2 diabetes; CKD, chronic kidney disease; HR, hazard ratio; CI, confidence interval; Ref, reference.

**Table S10. Associations of childhood-to-adulthood body size trajectories with disease incidence and mortality outcomes** **after multiple imputation of missing covariates.**

|  | **First component disease**  **HR (95% CI)** | **Double CRMM**  **HR (95% CI)** | **CVD-T2D**  **HR (95% CI)** | **CVD-CKD**  **HR (95% CI)** | **T2D-CKD**  **HR (95% CI)** | **Triple CRMM**  **HR (95% CI)** | **All-cause Mortality**  **HR (95% CI)** |
| --- | --- | --- | --- | --- | --- | --- | --- |
| **Body size trajectories** |  |  |  |  |  |  |  |
| Thinner-to-normal | 1.10 (1.06, 1.14) | 1.18 (1.07, 1.30) | 1.36 (1.17, 1.58) | 1.05 (0.92, 1.20) | 1.38 (1.11, 1.70) | 1.34 (0.96, 1.87) | 0.98 (0.94, 1.02) |
| Thinner-to-overweight | 1.62 (1.58, 1.67) | 2.21 (2.04, 2.40) | 3.13 (2.77, 3.53) | 1.58 (1.41, 1.77) | 2.89 (2.42, 3.45) | 3.01 (2.29, 3.96) | 0.91 (0.87, 0.95) |
| Thinner-to-obesity | 2.74 (2.65, 2.83) | 5.00 (4.61, 5.42) | 7.53 (6.66, 8.50) | 2.88 (2.56, 3.24) | 9.07 (7.64, 10.75) | 9.06 (6.94, 11.83) | 1.19 (1.14, 1.25) |
| Average-to-normal | Ref | Ref | Ref | Ref | Ref | Ref | Ref |
| Average-to-overweight | 1.40 (1.36, 1.44) | 1.79 (1.66, 1.93) | 2.26 (2.00, 2.55) | 1.50 (1.35, 1.66) | 2.17 (1.83, 2.59) | 2.35 (1.79, 3.07) | 0.91 (0.88, 0.95) |
| Average-to- obesity | 2.38 (2.31, 2.45) | 4.11 (3.80, 4.43) | 6.21 (5.52, 6.98) | 2.56 (2.30, 2.86) | 7.08 (5.99, 8.36) | 7.61 (5.87, 9.87) | 1.09 (1.04, 1.13) |
| Plumper-to-normal | 0.97 (0.91, 1.03) | 0.82 (0.68, 1.00) | 0.86 (0.64, 1.17) | 0.77 (0.59, 1.00) | 0.96 (0.64, 1.46) | 0.84 (0.42, 1.69) | 1.13 (1.06, 1.22) |
| Plumper-to-overweight | 1.44 (1.39, 1.50) | 1.91 (1.73, 2.11) | 2.35 (2.02, 2.73) | 1.56 (1.35, 1.79) | 2.59 (2.09, 3.20) | 2.48 (1.77, 3.48) | 1.01 (0.96, 1.06) |
| Plumper-to-obesity | 2.56 (2.48, 2.65) | 4.41 (4.06, 4.80) | 6.70 (5.91, 7.59) | 2.58 (2.27, 2.92) | 7.82 (6.56, 9.33) | 7.99 (6.07, 10.53) | 1.22 (1.16, 1.28) |

HRs and 95% CIs were estimated using Cox proportional hazards models based on five multiply imputed datasets generated by multiple imputation by chained equations (MICE). Estimates were pooled using Rubin’s rules. Models were adjusted for age, sex, educational attainment, ethnicity and Townsend Deprivation Index, family history of CVD, family history of T2D, polygenic risk score for BMI and LE4 score. LE4 indicates 4 lifestyle metrics from Life’s Essential 8. First component disease denotes the first occurrence of any one of CVD, T2D, or CKD during follow-up. Double CRMM denotes the first attainment of any two of these conditions (i.e., T2D-CVD, CVD-CKD, or T2D-CKD); the event date is the date of the second qualifying diagnosis. Triple CRMM denotes the occurrence of all three conditions; the event date is the date of the third qualifying diagnosis.

Abbreviations: CRMM, cardio-renal-metabolic multimorbidity; CVD, cardiovascular disease; T2D, type 2 diabetes; CKD, chronic kidney disease; HR, hazard ratio; CI, confidence interval; Ref, reference.

**Table S11. Associations of childhood-to-adulthood body size trajectories with disease incidence and mortality outcomes** **without adjustment for polygenic risk score for BMI (BMI-PRS).**

|  | **First component disease**  **HR (95% CI)** | **Double CRMM**  **HR (95% CI)** | **CVD-T2D**  **HR (95% CI)** | **CVD-CKD**  **HR (95% CI)** | **T2D-CKD**  **HR (95% CI)** | **Triple CRMM**  **HR (95% CI)** | **All-cause Mortality**  **HR (95% CI)** |
| --- | --- | --- | --- | --- | --- | --- | --- |
| **Body size trajectories** |  |  |  |  |  |  |  |
| Thinner-to-normal | 1.10 (1.06, 1.13) | 1.18 (1.07, 1.30) | 1.36 (1.17, 1.57) | 1.06 (0.93, 1.21) | 1.39 (1.13, 1.72) | 1.33 (0.95, 1.86) | 0.98 (0.94, 1.02) |
| Thinner-to-overweight | 1.63 (1.58, 1.68) | 2.22 (2.05, 2.41) | 3.14 (2.78, 3.55) | 1.60 (1.43, 1.79) | 3.00 (2.51, 3.57) | 3.03 (2.30, 3.99) | 0.91 (0.88, 0.95) |
| Thinner-to-obesity | 2.76 (2.68, 2.85) | 5.08 (4.69, 5.50) | 7.65 (6.77, 8.63) | 2.91 (2.59, 3.27) | 9.59 (8.09, 11.37) | 9.26 (7.10, 12.08) | 1.21 (1.15, 1.26) |
| Average-to-normal | Ref | Ref | Ref | Ref | Ref | Ref | Ref |
| Average-to-overweight | 1.40 (1.36, 1.44) | 1.80 (1.67, 1.95) | 2.27 (2.01, 2.56) | 1.50 (1.35, 1.67) | 2.22 (1.87, 2.64) | 2.37 (1.81, 3.10) | 0.92 (0.89, 0.95) |
| Average-to- obesity | 2.40 (2.33, 2.47) | 4.17 (3.86, 4.50) | 6.30 (5.60, 7.09) | 2.58 (2.31, 2.87) | 7.42 (6.28, 8.76) | 7.77 (6.00, 10.07) | 1.10 (1.06, 1.15) |
| Plumper-to-normal | 0.97 (0.92, 1.04) | 0.83 (0.68, 1.01) | 0.87 (0.64, 1.18) | 0.77 (0.59, 1.01) | 0.98 (0.65, 1.48) | 0.85 (0.42, 1.70) | 1.14 (1.06, 1.22) |
| Plumper-to-overweight | 1.45 (1.40, 1.51) | 1.93 (1.74, 2.13) | 2.37 (2.04, 2.76) | 1.57 (1.36, 1.81) | 2.67 (2.16, 3.31) | 2.52 (1.80, 3.52) | 1.02 (0.97, 1.07) |
| Plumper-to-obesity | 2.59 (2.51, 2.68) | 4.50 (4.14, 4.89) | 6.83 (6.03, 7.73) | 2.60 (2.30, 2.95) | 8.29 (6.95, 9.88) | 8.20 (6.24, 10.78) | 1.24 (1.18, 1.30) |

Models were adjusted for age, sex, educational attainment, ethnicity and Townsend Deprivation Index, family history of CVD, family history of T2D and LE4 score. LE4 indicates 4 lifestyle metrics from Life’s Essential 8. First component disease denotes the first occurrence of any one of CVD, T2D, or CKD during follow-up. Double CRMM denotes the first attainment of any two of these conditions (i.e., T2D-CVD, CVD-CKD, or T2D-CKD); the event date is the date of the second qualifying diagnosis. Triple CRMM denotes the occurrence of all three conditions; the event date is the date of the third qualifying diagnosis.

Abbreviations: CRMM, cardio-renal-metabolic multimorbidity; CVD, cardiovascular disease; T2D, type 2 diabetes; CKD, chronic kidney disease; HR, hazard ratio; CI, confidence interval; Ref, reference.

**Figure S1. Flow diagram of the study population selection.**

Abbreviations: CVD, cardiovascular disease; T2D, type 2 diabetes; CKD, chronic kidney disease; BMI, body mass index; LE4 indicates 4 lifestyle metrics from Life’s Essential 8. Component diseases refer to CVD, T2D, and CKD.

**Figure S2. Mediation effects of CTI on the associations between childhood-to-adulthood body size trajectories and the onset of different subtypes of CRMM.**

The figure shows the estimated total effects, ADE, and ACME of CTI on the associations of childhood-to-adulthood body size trajectories (Thinner-to-Obesity, Average-to-Obesity, and Plumper-to-Obesity vs Average-to-Normal) with different subtypes of CRMM, including T2D-CVD, CVD-CKD, T2D-CKD. Proportion mediated and corresponding P values are presented. All models were adjusted for age, sex, educational attainment, ethnicity and Townsend Deprivation Index, family history of CVD, family history of T2D, LE4 score and polygenic risk score for BMI. LE4 indicates 4 lifestyle metrics from Life’s Essential 8.

Abbreviations: CVD, cardiovascular disease; T2D, type 2 diabetes; CKD, chronic kidney disease; BMI, body mass index; LE4 indicates 4 lifestyle metrics from Life’s Essential 8.

**Figure S3. Age-stratified associations of childhood-to-adulthood body size trajectories with transitions along the comorbidity-count progression pathway (Healthy → First component disease → Double CRMM → Triple CRMM → Death) in multi-state models.**

Models were adjusted for sex, educational attainment, ethnicity and Townsend Deprivation Index, family history of cardiovascular diseases, family history of diabetes, LE4 score and polygenic risk score for BMI. LE4 indicates 4 lifestyle metrics from Life’s Essential 8. NE = not estimable due to sparse events or (quasi-)complete separation in the Cox model.

Abbreviation: CRMM, cardio-renal-metabolic multimorbidity.

**Figure S4. Sex-stratified associations of childhood-to-adulthood body size trajectories with transitions along the comorbidity-count progression pathway (Healthy → First component disease → Double CRMM → Triple CRMM → Death) in multi-state models.**

Models were adjusted for age, educational attainment, ethnicity and Townsend Deprivation Index, family history of cardiovascular diseases, family history of diabetes, LE4 score and polygenic risk score for BMI. LE4 indicates 4 lifestyle metrics from Life’s Essential 8. NE = not estimable due to sparse events or (quasi-)complete separation in the Cox model.

Abbreviation: CRMM, cardio-renal-metabolic multimorbidity.

**Figure S5. Age-stratified associations of childhood-to-adulthood body size trajectories with transitions along the comorbidity-pattern progression pathway in multi-state models.**

Models were adjusted for sex, educational attainment, ethnicity and Townsend Deprivation Index, family history of CVD, family history of diabetes, LE4 score and polygenic risk score for BMI. LE4 indicates 4 lifestyle metrics from Life’s Essential 8. NE = not estimable due to sparse events or (quasi-)complete separation in the Cox model.

Abbreviations: CVD, cardiovascular disease; T2D, type 2 diabetes; CKD, chronic kidney disease; CRMM, cardio-renal-metabolic multimorbidity.

**Figure S6. Sex-stratified associations of childhood-to-adulthood body size trajectories with transitions along the comorbidity-pattern progression pathway in multi-state models.**

Models were adjusted for age, educational attainment, ethnicity and Townsend Deprivation Index, family history of CVD, family history of diabetes, LE4 score and polygenic risk score for BMI. LE4 indicates 4 lifestyle metrics from Life’s Essential 8. NE = not estimable due to sparse events or (quasi-)complete separation in the Cox model.

Abbreviations: CVD, cardiovascular disease; T2D, type 2 diabetes; CKD, chronic kidney disease; CRMM, cardio-renal-metabolic multimorbidity.

**Figure S7. Associations of childhood-to-adulthood body size trajectories with transitions along the comorbidity-count progression pathway (Healthy → First component disease → Double CRMM → Triple CRMM → Death) in multi-state models, excluding individuals with adult BMI< 18.5 kg/m² (N = 392,556).**

Models were adjusted for age, sex, educational attainment, ethnicity and Townsend Deprivation Index, family history of cardiovascular diseases, family history of diabetes, LE4 score and polygenic risk score for BMI. LE4 indicates 4 lifestyle metrics from Life’s Essential 8.

Abbreviation: CRMM, cardio-renal-metabolic multimorbidity.

**Figure S8. Associations of childhood-to-adulthood body size trajectories with transitions along the comorbidity-pattern progression pathway in multi-state models, excluding individuals with adult BMI< 18.5 kg/m² (N = 392,556).**

Models were adjusted for age, sex, educational attainment, ethnicity and Townsend Deprivation Index, family history of CVD, family history of diabetes, LE4 score and polygenic risk score for BMI. LE4 indicates 4 lifestyle metrics from Life’s Essential 8. NE = not estimable due to sparse events or (quasi-)complete separation in the Cox model.

Abbreviations: CVD, cardiovascular disease; T2D, type 2 diabetes; CKD, chronic kidney disease; CRMM, cardio-renal-metabolic multimorbidity.

**Figure S9. Associations of childhood-to-adulthood body size trajectories with transitions along the comorbidity-count progression pathway (Healthy → First component disease → Double CRMM → Triple CRMM → Death) in multi-state models, excluding events occurring within the first two years of follow-up (N = 385,114).**

Models were adjusted for age, sex, educational attainment, ethnicity and Townsend Deprivation Index, family history of cardiovascular diseases, family history of diabetes, LE4 score and polygenic risk score for BMI. LE4 indicates 4 lifestyle metrics from Life’s Essential 8.

Abbreviation: CRMM, cardio-renal-metabolic multimorbidity.

**Figure S10. Associations of childhood-to-adulthood body size trajectories with transitions along the comorbidity-pattern progression pathway in multi-state models,** **excluding events occurring within the first two years of follow-up (N = 385,114).**

Models were adjusted for age, sex, educational attainment, ethnicity and Townsend Deprivation Index, family history of CVD, family history of diabetes, LE4 score and polygenic risk score for BMI. LE4 indicates 4 lifestyle metrics from Life’s Essential 8. NE = not estimable due to sparse events or (quasi-)complete separation in the Cox model.

Abbreviations: CVD, cardiovascular disease; T2D, type 2 diabetes; CKD, chronic kidney disease; CRMM, cardio-renal-metabolic multimorbidity.

**Figure S11. Associations of childhood-to-adulthood body size trajectories with transitions along the comorbidity-count progression pathway (Healthy → First component disease → Double CRMM → Triple CRMM → Death) in multi-state models, excluding individuals missing covariates (N = 382,638).**

Models were adjusted for age, sex, educational attainment, ethnicity and Townsend Deprivation Index, family history of cardiovascular diseases, family history of diabetes, LE4 score and polygenic risk score for BMI. LE4 indicates 4 lifestyle metrics from Life’s Essential 8.

Abbreviation: CRMM, cardio-renal-metabolic multimorbidity.

**Figure S12. Associations of childhood-to-adulthood body size trajectories with transitions along the comorbidity-pattern progression pathway in multi-state models, excluding individuals missing covariates (N = 382,638).**

Models were adjusted for age, sex, educational attainment, ethnicity and Townsend Deprivation Index, family history of CVD, family history of diabetes, LE4 score and polygenic risk score for BMI. LE4 indicates 4 lifestyle metrics from Life’s Essential 8. NE = not estimable due to sparse events or (quasi-)complete separation in the Cox model.

Abbreviations: CVD, cardiovascular disease; T2D, type 2 diabetes; CKD, chronic kidney disease; CRMM, cardio-renal-metabolic multimorbidity.

**Figure S13. Directed acyclic graph (DAG) depicting the assumed causal relationships among BMI polygenic risk score, childhood-to-adulthood body size trajectories, covariates, and cardio-renal-metabolic multimorbidity.**

Directed acyclic graph illustrating the assumed causal relationships between genetic susceptibility (BMI polygenic risk score, BMI-PRS), childhood-to-adulthood body size trajectory, sociodemographic factors, lifestyle factors (Life’s Essential 4), family history, and cardio-renal-metabolic multimorbidity. BMI-PRS may influence both body size trajectory and disease risk, representing shared genetic susceptibility.
